# A Wearable Lower Limb Exoskeleton: Reducing the Energy Cost of Human Movement

**DOI:** 10.3390/mi13060900

**Published:** 2022-06-06

**Authors:** Xinyao Tang, Xupeng Wang, Xiaomin Ji, Yawen Zhou, Jie Yang, Yuchen Wei, Wenjie Zhang

**Affiliations:** 1 School of Mechanical and Precision Instrument Engineering, Xi’an University of Technology, Xi’an 710048, China; tan_xiaoyao@outlook.com (X.T.); jixm@xaut.edu.cn (X.J.); 2 Research Center for Civil-Military Integration and Protection Equipment Design Innovation, Xi’an University of Technology, Xi’an 710054, China; 2200620004@stu.xaut.edu.cn (Y.Z.); 2200621043@stu.xaut.edu.cn (J.Y.); 2200621047@stu.xaut.edu.cn (Y.W.); 2200621044@stu.xaut.edu.cn (W.Z.)

**Keywords:** lower limb exoskeleton, wearable device, assisted movement, metabolic cost

## Abstract

Human body enhancement is an interesting branch of robotics. It focuses on wearable robots in order to improve the performance of human body, reduce energy consumption and delay fatigue, as well as increase body speed. Robot-assisted equipment, such as wearable exoskeletons, are wearable robot systems that integrate human intelligence and robot power. After careful design and adaptation, the human body has energy-saving sports, but it is an arduous task for the exoskeleton to achieve considerable reduction in metabolic rate. Therefore, it is necessary to understand the biomechanics of human sports, the body, and its weaknesses. In this study, a lower limb exoskeleton was classified according to the power source, and the working principle, design idea, wearing mode, material and performance of different types of lower limb exoskeletons were compared and analyzed. The study shows that the unpowered exoskeleton robot has inherent advantages in endurance, mass, volume, and cost, which is a new development direction of robot exoskeletons. This paper not only summarizes the existing research but also points out its shortcomings through the comparative analysis of different lower limb wearable exoskeletons. Furthermore, improvement measures suitable for practical application have been provided.

## 1. Introduction

In recent years, wearable exoskeleton devices have widespread development in medical rehabilitation, assisted movement and military fields, which are widely used in scientific research, industrial production and daily life [1,2,3]. In the field of medical rehabilitation, assisted exoskeletons are mainly used in basic rehabilitation training, disabled walking, medical care, and other aspects. They are used as an auxiliary rehabilitation device to carry out rehabilitation training for patients. In the military field, with the birth of weapons of mass destruction, the physical fitness and load of soldiers in extreme environments have become the weak points in military development. Therefore, all countries are trying to increase the survival and load capacity of soldiers in order to improve the combat ability of individual soldiers. In the civil field, the booster exoskeleton is mostly used for firefighting, transportation, and other work to reduce the burden on the human body.

An exoskeleton can be classified as enhancing human abilities, such as load capacity or walking speed, or improving human endurance by reducing the metabolic needs of a given activity. In most of the literature, the ultimate goal of the exoskeleton is to reduce muscle activation and increase endurance during running, but the difference between the two types of devices is often blurred. For example, a device designed to reduce the metabolic demand of exercise can also perform the exercise at a higher speed for a given metabolic demand. In addition, some orthopedic devices designed to restore lost function may be considered exoskeletons. Therefore, the exoskeleton can be roughly divided into three categories according to the use of power, which are passive exoskeleton, quasi-passive exoskeleton, and powered exoskeleton regarded as active equipment [4,5]. According to the human body parts supported by an exoskeleton, they can be divided into upper limb exoskeleton, lower limb exoskeleton (lees), whole-body exoskeleton, and specific joint-supporting exoskeleton [6,7,8,9,10,11,12,13]. These exoskeleton systems are divided into three categories according to their different applications and target users, which are gait rehabilitation, human movement, and human strength enhance [14].

The passive exoskeleton adds a power source. A powered exoskeleton is a man-machine coupling assisted device. Biomechanical factors, sensors, drivers, control systems, and exoskeleton systems should be considered in the design [15,16,17]. It has higher requirements in man-machine coordination, and its working principle must be guaranteed to meet the physiological structure and operating mechanism of the human body as much as possible [18]. In addition, the sensing and control method, modeling stability, and comfort of a lower limb exoskeleton will also affect its performance [19]. The main function of an unpowered exoskeleton is to convert the human body’s own gravity potential energy, motion energy, or external load to the energy storage element in order to replace the bearing force of human bones and joints. In particular, when the human body has to bear huge impact force, most of the force is absorbed by unpowered exoskeleton.

This paper mainly studies the wearable lower-limb exoskeleton system and discusses the typical lower limb exoskeletons that have been developed all over the world. The working principle, design idea, wearing mode, material, and performance of different types of lower limb exoskeleton are examined. Through comparative analysis of these aspects, the improvement measures suitable for practical application are proposed. Finally, the limitations of the existing wearable lower limb exoskeletons and the development directions of related research are described.

## 2. Analysis of Lower Limb Movement Characteristics

In daily life, the movement of human lower limbs has a strong periodicity. The common forms of movement are walking, running, jumping, etc., and the degrees of freedom of lower limbs are mainly distributed in the hip, knee and ankle [20]. Walking gait is closely related to changes in joint force, torque, and angle [21].

### 2.1. Range of Joint Motion

The maximum range of motion of lower limb joints and the range of normal walking motion refer to the normal range of motion of human joints [16], as shown in Table 1. The human body can be calibrated with three mutually perpendicular basic planes, namely, sagittal, frontal, and horizontal. In the normal walking process of the human body, the walking movement mainly occurs in the sagittal plane, and there is little to no movement in the frontal plane and horizontal plane. Therefore, when designing a lower limb assisted exoskeleton robot, the flexion/extension motion of each joint is the main consideration goal, and the motion limit of the corresponding degree of freedom should be greater than the motion range of the joint. However, when analyzing the actual movement of human body, we need to comprehensively consider three fundamentals.

### 2.2. Gait Characteristics Based on Plantar Pressure

Gait parameters are usually divided into four types: space-time (time and distance), kinematic (joint displacement, angle, velocity, acceleration and other parameters), dynamic (joint force, torque, ground reaction and other parameters), and biological (muscle force and EMG signal parameters). At present, researchers usually use statistical methods to summarize a reference range for gait parameters such as stride time, motion angle range, and joint torque in healthy people of different ages and genders as gait law [22,23,24]. In the process of walking, the human body constantly does positive and negative work to overcome gravity. Wearing an exoskeleton may lead to changes in these gait parameters.

**Figure 1:**
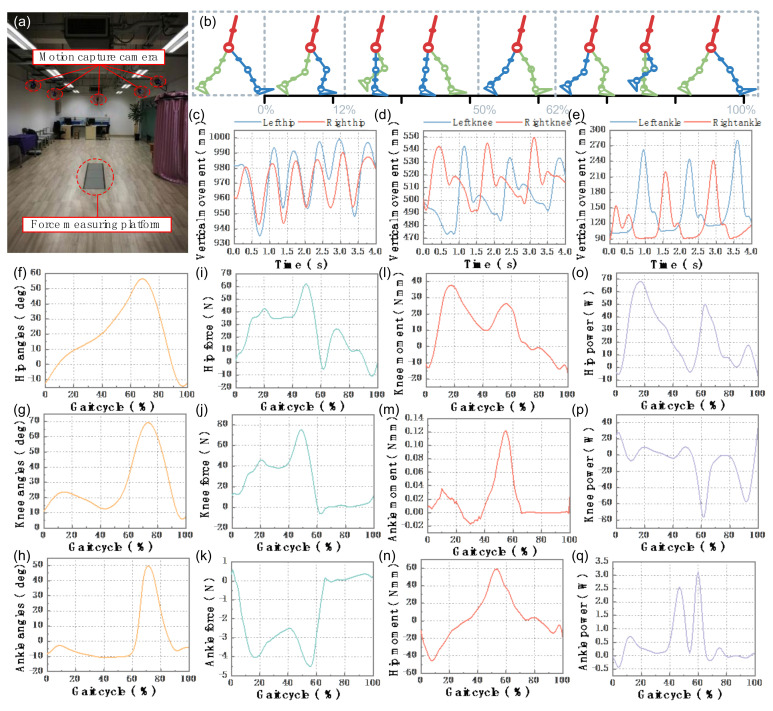
The periodic movement of lower limb joints. (**a**) Experimental scene. (**b**) Gait cycle. (**c**–**e**) show the vertical alternating movement of the left and right hips, knees and ankles over a period of 4 seconds. (**f**–**h**) show the hip, knee and ankle angles during the gait cycle. (**i**–**k**) show the hip, knee and ankle forces during the gait cycle. (**l**–**n**) show the hip, knee and ankle moments during the gait cycle. (**o**–**q**) show the hip, knee and ankle power during the gait cycle.

The variation characteristics of plantar contact pressure [25] can be described as initial contact phase (ICP), rear foot contact phase (RFCP), mid foot contact phase (MFCP), fore foot contact phase (FFCP), and total foot contact phase (TFCP). The gait data on human lower limb walking movement are obtained using a Vicon motion capture system and an AMTI force measuring platform (Figure 1a). It is found that the changes of joint angle, joint force, and joint torque are periodic [26], and the joint angle curves under different walking speeds have different periods and similar shapes [27] (Figure 1c–e). In the process of walking, it is called a gait cycle from the heel touchdown in the standing posture stage to the heel touchdown again (Figure 1b). As shown in Figure 1f–q, the angle, force, and moment, as well as power changes of the hip, knee, and ankle in a gait cycle are shown.

### 2.3. Assisted Movement Mode

**Figure 2:**
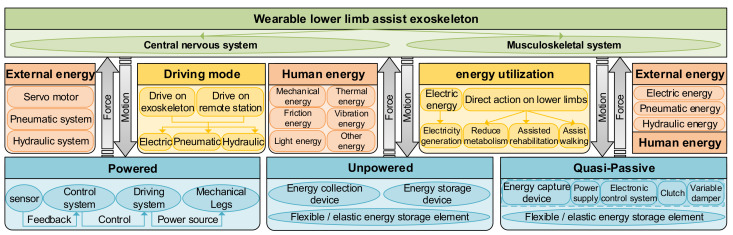
Characteristics of wearable exoskeletons.

When the human body consumes more metabolic energy, people feel tired. However, by reducing metabolic energy, people can improve their work efficiency and break through the exercise limit of the human body, so there is the generation of lower limb-assisted exoskeletons [28,29]. According to the power sources, the lower limb assist exoskeleton can be divided into powered lower-limb exoskeleton, unpowered lower limb exoskeleton, and quasi-passive lower limb exoskeleton. The characteristics of wearable exoskeletons are shown in Figure 2.

Powered lower limb exoskeleton. Electric kinetic energy [30], pneumatic kinetic energy [31,32], hydraulic energy [33], and other forms of energy are used as power sources to input them into the energy system of the lower limbs. According to the law of energy conservation, additional energy is injected into the lower limbs of the human body, resulting in the reduction of the initial input value of metabolic energy, so the metabolic energy of the lower limbs consumed by the human body is reduced.

Unpowered lower limb exoskeleton. By using people’s own metabolic energy and driving the joint movement of lower limbs with the help of an elastic energy storage mechanism, part of human energy is transformed into mechanical energy, thermal energy, friction energy, and vibration energy; collected; and fed back to the lower limbs. According to the law of energy conservation, part of the energy that should have been consumed is recycled to the system, so the metabolic energy of the system is reduced, which plays a role in helping the movement of the lower limbs and improving the energy utilization rate [28,34,35].

Quasi-passive lower limb exoskeleton. The support torque is provided by the passive element, and the support is engaged or separated by the small actuator, thus forming a quasi-passive device. The actuator used for this purpose is called the clutch elastic actuator (CEA) [36]. With passive support that makes it possible to engage or separate exoskeletons, users can achieve unobtrusive movement in tasks that do not require help.

In short, the powered exoskeleton needs to carry an energy storage module, which is heavy, and the operation time is limited. Additional loads produce system inertia that is difficult to compensate for [37]. In addition, unpowered exoskeleton systems have been proved to reduce muscle activity and fatigue, so as to reduce injury and the risk of musculoskeletal diseases [38,39,40,41]. However, their versatility is usually limited because some mobility can be limited when users do not need support, such as for walking of the spinal exoskeleton [42] and arm movement of the shoulder exoskeleton. The use of quasi-passive devices can solve this problem, and the controller can decide when to open and close the exoskeleton.

## 3. Wearable Lower Limb Exoskeletons for Various Assisted Movement

### 3.1. Powered Lower Limb Assisted Exoskeleton

Development began on active lower limb assist exoskeletons, namely, powered lower limb assist exoskeletons, more than 70 years ago. In terms of time, the former Soviet Union and the former Yugoslavia developed the earliest, and the European American and Japan developed the fastest. In terms of application fields, these exoskeletons were applied in military research earlier, and the technology is relatively advanced. They are also used for healthy people to load, enhance human strength, and reduce the metabolic energy of the lower limbs. They also help people with dyskinesia to carry out basic daily activities. The research on these aspects is relatively late, and the technology is relatively backward.

In the past 20 years, thanks to the development of new technologies and components such as high-performance processors and sensors, the research and development of assisted exoskeleton robots has been successful [43]. In particular, the United States, Japan, and some European countries have made outstanding achievements in the research and development of exoskeleton robots and gradually achieved practical application [44].

#### 3.1.1. Multi Joint Assisted Exoskeleton

The exoskeleton first appeared in 1948, when Professor Bernstein from Moscow in the former Soviet Union designed the world’s first motor-driven lower limb exoskeleton system. In 1971, Professor Vukobratovic from the former Yugoslavia developed the first cylinder-driven lower extremity exoskeleton for patients with spinal cord injury [45]. With the maturity of related technologies, lower limb exoskeletons developed rapidly at the end of the 20th century.

**Figure 3:**
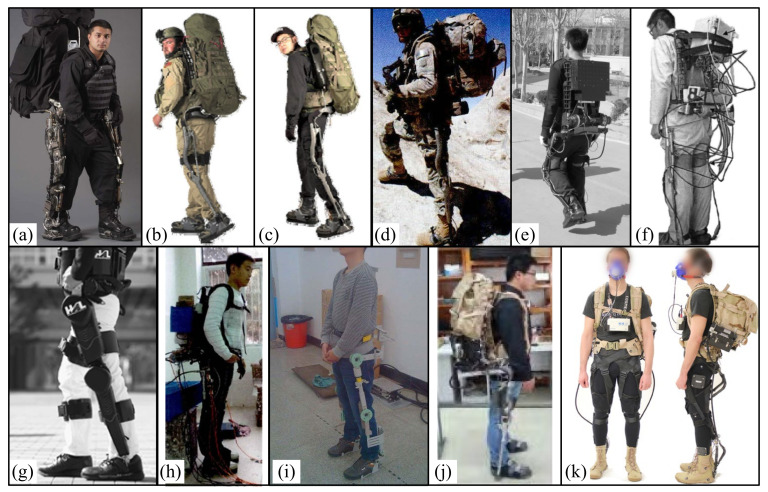
Multi-joint powered lower limb assisted exoskeleton. (**a**) University of California, BLEEX [8]. (**b**) ExoHIKE [48]. (**c**) ExoClimber [48]. (**d**) Lockheed Martin, HULC [43]. (**e**) Naval Academy of Aeronautical Engineering, the third-generation prototypes [50]. (**f**) East China University of Technology, ELEBOT [52]. (**g**) University of Tsukuba, HAL-5 [59]. (**h**) Southwest Jiaotong University, 2012 [63]. (**i**) Harbin Institute of Technology, 2013 [61]. (**j**) Beijing University of technology [63]. (**k**) Harvard University, MJSE [65].

In 2004, the University of California designed the first lower limb exoskeleton (BLEEX) (Figure 3a), which was developed to help soldiers carry heavy objects. It belongs to the first field lower limb exoskeleton, which is composed of two powered humanoid legs, a power device and a backpack-like frame, on which various heavy objects can be installed [8,46]. Each leg has seven degrees of freedom: three degrees of freedom for the hip, one degree of freedom for the knee, and three degrees of freedom for the ankle. Among these degrees of freedom, hip flexion/extension, hip abduction/ adduction, knee flexion/ extension, and ankle dorsiflexion/plantar flexion are driven by linear hydraulic actuators, and the other degrees of freedom are driven passively by steel springs and elastomers. It is reported that BLEEX wearers walk at an average speed of up to 1.3 m/s with a 34 kg payload (exoskeleton weight + payload up to 75 kg in total). The team also developed several other exoskeletons: the ExoHIKE (Figure 3b) and ExoClimber (Figure 3c), a HULC exoskeleton (Figure 3d), and a human universal carrier [47].

In 2006, the human universal load carrier (HULC) (Figure 3d) was launched by Berkeley bionics. It has two independent features: (1) increasing the maximum load-bearing capacity of the wearer (150 to 200 pounds) and (2) reducing the metabolic cost of the wearer. In some preliminary evaluations in late 2006 and early 2007, when users walked at 2 mph with the latest exoskeleton (without payload), the oxygen consumption was reduced by 5~12%. When the user carries the load, the effect is more obvious. When these users with exoskeletons carried 81 pounds at a speed of 2 mph, their oxygen consumption reduced by about 15%. This is the first exoskeleton in the world to demonstrate reduced oxygen consumption by users [48].

In 2007, the NAEIES exoskeleton [49,50] (Figure 3e) designed by the Naval Academy of Aeronautical Engineering was driven by motor and gas spring, and different control strategies were simulated. At present, three generations of prototypes have been designed. The third-generation exoskeleton is driven by air spring and cable motor, and some structures are made of carbon fiber. The mass of the prototype is 21.2 kg. The user can walk at the speed of 3.6 km/h for 2 h under the load of 300 N. The bionic design of the exoskeleton is good, and it can complete the actions of going upstairs and downstairs, squatting, and crawling forward.

In 2008, East China University of Science and Technology developed the ELEBOT exoskeleton prototype [51,52,53] (Figure 3f), which adopts hydraulic driving to realize various functions such as plantar pressure sensing and joint assistance. On this basis, its virtual prototype was optimized and improved. In 2014, Fang Mingzhou et al. simulated and optimized it [54]. The exoskeleton uses a lithium battery as the power source, adopts a hydraulic driving device, and relies on plantar pressure and powered sensors (angle sensors and force sensors) to obtain the wearer’s movement intention. The total weight is 25 kg. It realizes the functions of plantar pressure sensing and joint assistance, and it can provide continuous power of at least 35 kg load for 12 h.

In 2009, Lockheed Martin obtained the license to sell this concept to the U.S. military. The new HULC is a powered lower limb assisted exoskeleton that was developed and developed by Lockheed Martin in 2017. The product comes from Berkeley Robotics and Human Engineering Laboratory (BRHEL) and is designed to help soldiers walk as long as possible while wearing combat equipment [48,55]. In 2009, Honda developed a “powered lower limb assisted exoskeleton with weight support”, which can reduce the reaction force of the ground to users and ultimately reduce the leg muscle activity and whole-body energy consumption of users when climbing stairs or squatting [56].

In 2009, the hybrid assisted limb (HAL) was developed by the University of Tsukuba, Tsukuba, Japan, in order to help healthy people, enhance their strength and help people with dyskinesia carry out basic daily life exercises [57,58]. HAL has three configurations, whole-body, two legged, and one legged. The fifth version of HAL (HAL-5) is a whole-body exoskeleton used to enhance the strength and rehabilitation of healthy individuals [59]. HAL-5 (Figure 3g) weighs about 23 kg, and 15 kg is worn on the lower body. HAL-5 has eight controllable joints, including lower limb joints and upper limb joints, that are driven by motors. It can help people grasp heavy objects weighing up to 70 kg and play an auxiliary role in emergency rescue.

In 2012, Southwest Jiaotong University developed a hydraulic-driven lower limb exoskeleton experimental platform (Figure 3h) and studied its dynamic characteristics. The total mass of the exoskeleton is 30 kg, which can provide an additional 15 kg external load when wearing the device. During the test, the experimenter, as a support, carried part of the load, so that the actual load on the exoskeleton may not reach 45 kg [60].

In 2013, the State Key Laboratory of Robotics and Systems of Harbin University of technology developed a lower limb assisted exoskeleton device (Figure 3i) and used ADMAS to simulate and analyze the mechanism of the device. The knee and hip joints were driven by the motor to achieve assistance. When the load is 40 kg, the contact force between the exoskeleton and the wearer is very small. At the same time, the maximum contact force between the exoskeleton and the wearer should not exceed 45 N, about 4 kg [61,62].

In 2016, the weight-bearing exoskeleton [63,64] (Figure 3j) designed by Beijing University of Technology has been developed to the third generation of lower limb weight-bearing exoskeleton prototype, with a mass of 16kg. The flexion/extension of hip and knee joints is driven by hydraulic pressure and powered by two lithium batteries, which can ensure a load of 45 kg and walking for one hour at 4 km/h. Moreover, under the condition of load, it can complete the actions of going up and down stairs, going up and down slopes, walking on the grass, and so on.

In 2016, Harvard University designed a bionic multi-joint soft suit (Figure 3k) that can reduce the energy consumption of weight-bearing walking. It is composed of a belt, bilateral thigh pieces, and bilateral calf belts, with a total mass of 6.6 kg. Among them, two drive units are installed on a backpack and connected to the external clothing through a Bowden cable. The drive retracts the Burton line to provide a controlled force for the wearer. The results show that external clothing can transmit controlled force to the wearer. The results showed that compared with unpowered removing the equivalent mass of the device (EXO_OFF_EMR) (7.9 ± 0.8 W/kg) and unpowered (EXO_OFF) (8.5 ± 0.9 W/kg), the net metabolic power under powered (EXO_ON) (7.5 ± 0.6W/kg) decreased by 7.3 ± 5.0% and 14.2 ± 6.1%, respectively. Compared with the EXO_OFF state, the average muscle activation rate of lateral femoral muscle in the EXO_OFF_EMR state decreased significantly (4.7 ± 7.0%), and the average muscle activation rate of EXO_ON flounder decreased significantly (8.4 ± 9.8%) [65].

#### 3.1.2. Hip Assisted Exoskeleton

**Figure 4:**
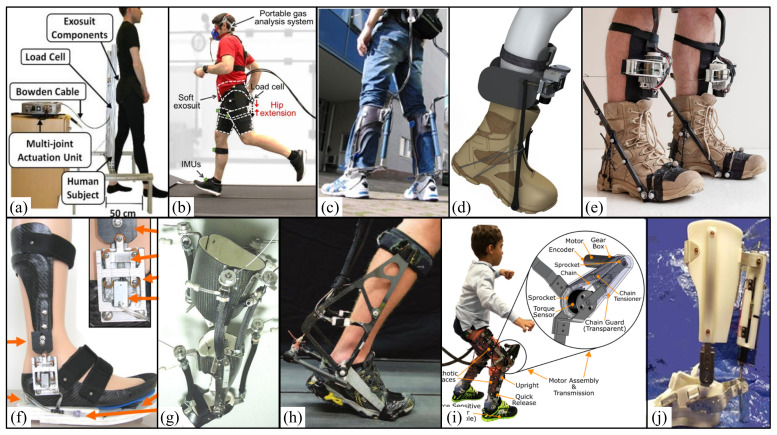
Single-joint powered lower limb assisted exoskeletons. (**a**) Harvard University, SE [66]. (**b**) Harvard University, TSE [67]. (**c**) Achilles exoskeleton [71]. (**d**) MIT, AAE [72]. (**e**) MIT, AAE [74]. (**f**) University of Illinois KFO [69]. (**g**) University of Michigan, KAFO [70]. (**h**) Carnegie Mellon University, AE [73]. (**i**) National Institutes of Health, RETCG [4]. (**j**) Arizona State University, DCO [68].

In 2016, Harvard University designed a soft suit for the hip joint (Figure 4a) that is a hip extension assisted iterative controller based on an inertial sensor. The controller is implemented on a single joint soft plug-in and coupled to a laboratory-based multi-joint driving platform that can quickly reconstruct the implementation of different sensors and control strategies. The results showed that the average transfer force started at the same time as the time of the maximum hip flexion angle and reached the peak time (expected to be 23%) after 22.7 ± 0.63% of the gait cycle, and the peak amplitude was 198.2 ± 1.6 N (expected 200 N), which was equivalent to the average peak torque of 30.5 ± 4.7Nm. When comparing powered and unpowered conditions, metabolic reduction was found to range from 5.7% to 8.5% [66].

In 2017, Harvard University designed a tether soft suit for the hip joint (Figure 4b). When participants run in the soft suit, the outboard drive system generates auxiliary force, and a Bowden cable transmits the force to the suit. External force (load cell), segmental motion (IMUs), ground reaction force (Instrumental treadmill) and metabolic rate (indirect calorimetry) were measured. The experimental results show that the hip extension assistance of the tethered soft outer clothing can reduce the metabolic cost of running by 5.4% compared with that without outer clothing. The ultimate goal of this study is to develop a wearable system containing all driving and control hardware, with an estimated mass of less than 5 kg [67].

#### 3.1.3. Knee Assisted Exoskeleton

In 2016, the National Institutes of Health introduced the design and preliminary results of a dynamic exoskeleton (Figure 4i) that is used to treat the flexion gait of children with cerebral palsy (CP). The device was lightweight (3.2 kg) and modular and was tested on a 6-year-old male participant with spastic diplegia. The results showed that the dynamic exoskeleton increased the extension of the knee joint by 18.1° and the total range of motion of the knee joint by 21.0°. The experimental results show that there is no significant reduction in knee extensor activity, so users do not just rely on the exoskeleton to stretch their limbs. These results preliminarily confirmed the use of a robot exoskeleton in the treatment of squatting posture [4].

#### 3.1.4. Ankle Assisted Exoskeleton

In 2007, Arizona State University designed a portable dynamically controlled ankle-foot orthosis (DCO) (Figure 4j) [68] that is composed of a small DC motor, an efficient lead screw, and a spring. The tendon mass is 0.95 kg. By using the elastic energy potential in the unique tuned coil spring to obtain power, power amplification of 2.38 is realized. The motor provides 55W, and the spring provides the remaining 76 W. The test results with two healthy subjects show that the power amplification increases by twice the current amount, indicating that the mechanical system and energy consumption can be reduced to the wearable level. In addition, the new controller can start, stop, and adjust the gait speed on the treadmill. Three healthy people and two subjects with gait defects were tested for several months. The results show that the control method is sufficient for clinical application.

In 2009, the University of Illinois developed and tested lightweight carbo-fiber ankle foot orthosis (AFO) [69]. It has artificial pneumatic muscles and can provide power for plantar flexion and dorsal flexion of the ankle joint when walking (Figure 4f). Then, the University of Michigan designed a unilateral dynamic knee-ankle-foot orthosis (KAFO) (Figure 4g) with antagonistic artificial pneumatic muscle pairs at the ankle (i.e., plantar flexor and dorsal flexor) and knee (i.e., extensor and flexor) [70]. The exoskeleton uses the user’s own surface EMG record to control the pneumatic muscle of the orthosis.

In 2013, the University of Bergen in the Netherlands designed an exoskeleton to help plantar flexion that can pneumatically control walking and help plantar flexion through different driving times. The study found that starting the exoskeleton before the contralateral leg contacts the heel can reduce the metabolic cost by 0.18 ± 0.06 w/kg, 6 ± 2% lower than the cost of walking without exoskeleton, and the soleus EMG activation is reduced by about 36% [35].

In 2014, the Achilles exoskeleton (Figure 4c) was developed that is a powered lower limb assist exoskeleton developed by Cor Meijneke et al. As a representative powered lower limb assisted exoskeleton, it can help human lower limbs reduce metabolic energy with a series elastic mechanism (SEA) with a carbon-fiber-reinforced leaf spring as a lever arm composed of a motor and ball screw gear. The spring energy storage mechanism serves as a kind of “pneumatic muscle”. At the same time, the dynamic model including motor and gear, spring stiffness, and exoskeleton structure was designed, and the design parameters were optimized and could generate 52% of the usual plantar bending force around the ankle. The equipment itself is smaller, which ensures the safety and comfort of the equipment to the greatest extent and improves the working efficiency of the human body [71].

In 2014, a lower limb assisted exoskeleton that could autonomously reduce the metabolic energy of human lower legs during walking was developed by Mooney et al. at the Massachusetts Institute of Technology [72] (Figure 4d). The device is a power-assisted exoskeleton device acting on the ankle, including a motor, a gear, a flexible wire, and a connecting rod mechanism connected with wearable shoes. The device is a power-assisted exoskeleton device acting on the ankle, including a motor, a gear, a flexible wire and a connecting rod mechanism connected with wearable shoes to match the periodic changes of the ankle during walking. When the joint does positive work, the connecting rod mechanism and flexible rope store the mechanical energy of the joint. When the joint does negative work, the motor converts electrical energy into mechanical energy. In the standing stage, the device provides a considerable level of positive mechanical energy for the ankle. The researchers measured the metabolic energy expenditure of seven subjects walking on a horizontal treadmill at a speed of 1.5 m/s wearing a 23 kg vest. The test experiments involved a non-wearing exoskeleton, a dynamic wearing exoskeleton, and a non-dynamic wearing exoskeleton. The experimental results show that the use of a leg exoskeleton can significantly reduce the metabolic cost of walking by about 36 ± 12 W. Additionally, the energy consumption decreased by 8 ± 3% compared with the control condition without the exoskeleton.

In 2015, Carnegie Mellon University designed two types of ankle exoskeleton, among which Alpha exoskeleton (Figure 4h) adopts a leaf spring as a series of elastic elements and lever arm; this unit has stronger adaptability, was cheaper to manufacture, had a lighter structure in the uncontrolled direction, and weighed less than 0.87 kg. The ankle exoskeleton end effector is driven by a powerful outboard motor and a real-time controller. The mechanical power is transmitted through the flexible Bowden cable, which is designed to help human users and reduce the overall energy cost. However, placing the ankle exoskeleton on the leg will automatically lead to the loss of metabolic energy because it will increase the distal weight. Reducing the total mass of equipment helps to minimize the resulting loss. An ankle exoskeleton also interferes with natural movement. Although this problem can be partially solved through good control, some interference is inevitable due to the physical structure of the equipment [73].

In 2016, MIT designed an active autonomous ankle exoskeleton (Figure 4e) with a total mass of 3.6 kg. It is driven by a winch drive, and the motor controller and battery are worn on the chest and waist. The experimental results show that the force generated by the active articular exoskeleton of each leg is 0.105 ± 0.008 w/kg. The walking cost with the active exoskeleton (3.28 ± 0.10 w/kg) was 11 ± 4% lower than that without the exoskeleton (3.71 ± 0.14 w/kg). With the wearing ankle exoskeleton, the average positive power of the ankle, knee, and hip decreased significantly, by 0.033 ± 0.006 w/kg, 0.042 ± 0.015 w/kg, and 0.034 ± 0.009 w/kg, respectively [74].

In conclusion, by analyzing exoskeletons with different driving modes, it is found that powered exoskeletons are more likely to be used for load bearing and output. The additional power source makes the mass of the dynamic exoskeleton very large. The dynamic exoskeleton performance is mainly affected by sensing and control methods, modeling stability, and comfort. From the perspective of assisted joints, the powered exoskeleton is more inclined to multi-joint assistance and is mainly used to improve the weight-bearing capacity. Ankle joint assistance is mostly used in single-joint assistance to assist walking, so as to reduce human energy consumption.

### 3.2. Unpowered Lower Limb Assisted Exoskeleton

Passive lower limb exoskeletons, that is, unpowered lower limb exoskeletons, are a new power-assisted system. According to the number of auxiliary lower limb joints, they can be divided into multi-joint passive lower limb and single-joint passive [75]. Compared with other countries’ exoskeleton assisted technology, American exoskeleton assisted technology has developed rapidly, from the development stage of “no” to a new stage of “excellent and excellent” [76]. At present, the military has invested a significant amount in this kind of exoskeleton to study new principles such as passive flexible/ elastic joints, bionic mechanical skeletons, and human energy collection devices, which are mainly used in medical rehabilitation assistance, human motion assistance, and load enhancement. For passive exoskeletons, lightweight design is more important. In order to reduce the energy consumption of the wearer, many different strategies have been adopted.

The idea of using exoskeletons to enhance human movement dates to 1890, when Nicholas Yagn conceived a device to promote walking, running, and jumping. The device consists of a huge bow spring that connects the belt and the foot accessories. Bow springs store body weight and energy generated by walking, running, or jumping. Yagen’s bow spring design lacks a certain degree of freedom in the knee. Therefore, in order to achieve a stable state, the user will jump from one leg to the other. Bending the knee during normal walking and running requires a great deal of energy to be stored in the bow spring. Yagen’s instrument is completely passive and driven by manpower [77].

#### 3.2.1. Multi-Joint Assisted Exoskeleton

**Figure 5:**
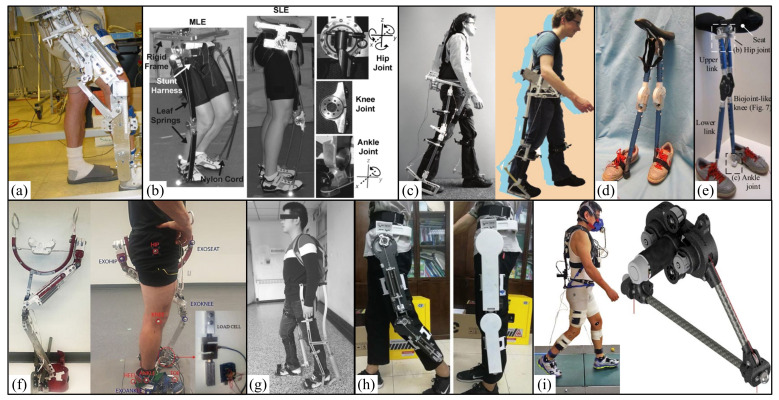
Multi-joint passive lower limb assisted exoskeletons. (**a**) University of Delaware, GBE [79]. (**b**) MIT, MLE, & SLE [81]. (**c**) Delft University of Technology, XPED1 & XPED2 [82,83]. (**d**) Zhejiang University, LEE [84]. (**e**) Huazhong University of science and technology, WSLEE [85]. (**f**) University of Ottawa, PWAE [34]. (**g**) Hebei University of Technology, NNLELE [88]. (**h**) Beijing University of Aeronautics and Astronautics, PLEE [89]. (**i**) Queen’s University of Canada, REE [90].

In 2006, the University of Delaware developed a gravity-balanced passive exoskeleton GBE for human legs (Figure 5a). It is composed of a rigid telescopic connecting rod, a rotating joint, and two springs that can be adjusted according to the geometry and inertia of the human leg wearing it. The device uses a parallelogram to determine the mass center of the mechanism and adds two springs to the mechanism, so that the total potential energy of the mechanism remains unchanged with the displacement of people. During the experiment, two groups of experiments were carried out: (1) Five healthy subjects were tested in the static configuration. Under the conditions of leg muscle relaxation, hip flexion (40~60°), and knee flexion (65~72°), the the correction and filtered electromyography (EMG) were compared of three muscles with and without equipment; (2) five healthy subjects and one patient (pre-stroke hemiplegia) walked on a treadmill to compare the effects of gravity alone in the case of leg and equipment gravity balance (leg and equipment balance) or only equipment gravity balance (equipment balance only). The experimental results showed that: (1) The percentage of IEMG (comprehensive muscle electromyography) in the balanced state of leg and instrument was always lower than that in the non-instrument state (*p* < 0.05); (2) when the tester walked at the optimal walking speed of 1 mile/h or 0.447 M/s, the hip and knee angles of healthy wearers increased by 22% and 24%, respectively; the hip and knee angles of stroke patients increased by 45% and 85%, respectively; and the step length increased by an average of 5.73% [78,79,80].

In 2009, MIT designed two elastic lower limb exoskeletons parallel to the legs that are respectively composed of multi-leaf (MLE) and single-leaf (SLE) glass fiber springs (Figure 5b) with masses of 6.75 kg and 6.26 kg, respectively. The jumping state of subjects wearing the exoskeleton is similar to that with a linear spring mass system, with the same total stiffness as that of normal jumping. At the same time, it reduces the force generated by the lower limbs and reduces the metabolic energy required for exercise. Nine subjects were selected for the experimental test. They jumped at 2.0 Hz, 2.2 Hz, 2.4 Hz, and 2.6 Hz with and without exoskeleton. At the same time, the ground reaction force, exoskeleton compression rate, and metabolic rate were measured. The experimental results show that wearing the multi-leaf spring reduces the net metabolic power by 6% on average, and wearing the single-leaf spring reduces the net metabolic power by 24% on average [81].

In 2011, Delft University of Technology in the Netherlands developed a lower limb exoskeleton XPED1 (Figure 5c) that is composed of an artificial tendon (an elastic element) and adjustable main frame with a mass of about 12 kg. The exoskeleton uses a passive structure called an artificial tendon that is installed parallel to the leg. It stores and redistributes the energy of human leg joints. In the exoskeleton performance evaluation experiment, 9 subjects tested energy consumption and muscle activation. The test experiments include normal walking, walking without the artificial tendon exoskeleton, and walking with the artificial tendon exoskeleton. It is evaluated from three aspects: (1) for artificial tendon strength: two load cells (Futek LTH 300, Irvine, CA, USA) are used to measure the tension of the artificial tendon; (2) for energy consumption: an open circuit respiratory measurement system is used to measure oxygen consumption and CO_2_ emission; (3) for muscle activity: in all experiments, the EMG of 8 groups of left leg muscles were recorded; (4) for heel position: reflection markers are used to record the positions of the two heels, and a motion capture system (Vicon, Oxford Metric Group, Oxford, UK) is used to track the position of the markers. The actual verification found that when walking with the exoskeleton, the energy consumption with artificial tendon was only reduced by 2.4% compared with that without artificial tendon [82].

In 2014, Delft University of Technology in the Netherlands designed another lower limb exoskeleton robot XPED2 (Figure 5c) that uses the mechanism of spring, cable, lever arm, and pulley to temporarily store and transfer energy between joints. The elasticity of the system is mainly composed of a customized glass fiber leaf spring and a unidirectional fiber ankle lever, with a total mass of 6.91 kg. In the experiment, five men and one woman were used to test. In order to minimize the impact of learning, three walking conditions were evaluated: no exoskeleton, no tension in the exoskeleton exotendon, and tension in the exoskeleton exotendon. All measurements were carried out at a fixed treadmill speed of 1 m/s; in the final stage, five walking tests were carried out: (1) two unsupported walks; (2) two supported walks; and (3) a baseline walk. The experimental results showed that: (1) the maximum ankle dorsiflexion angle decreased by 5.1° and the ankle plantar flexion torque increased by 0.022 Nm/kg with the exoskeleton compared with the condition without the exoskeleton; (2) when the exoskeleton tendon was tightened, the average joint torque decreased by 12.1%, but the average metabolic energy consumption increased by 6.1%; and (3) there was little difference in the kinematics and dynamics between hip and knee [83].

In 2015, Zhejiang University of China developed a lower limb knee exoskeleton (LEE) for weight-bearing support (Figure 5d) with a mass of 2.357 kg. Based on the gait of human lower limbs, it is divided into two parts according to the angle of knee joint: compliance coupling and weight support. The kinematics of the lower limbs and the LEE are numerically and experimentally analyzed to evaluate the design performance of the LEE, specifically by comparing the plantar force between the two gait patterns with and without the exoskeleton. The experimental results show that in a single gait cycle, the first peak and second peak of wearing the LEE are reduced by 27% and 21%, respectively, which verifies its support effect on human weight [84].

In 2016, Huazhong University of Science and Technology in China used a passive weight with flexible joints to support a lower limb exoskeleton (Figure 5e) to reduce the compression load of the knee joint, with a mass of 1 kg. The numerical simulation is carried out by COMSOL software. The results show that the lower limb exoskeleton based on gait reduces the plantar pressure, which proves its effectiveness in supporting the user’s weight when walking [85].

In 2019, the University of Ottawa in Canada developed a passive walking assisted exoskeleton (Figure 5f) that is composed of two support legs and seat frame units with a mass of 5.68 kg. When the weight of the user decreases the seat unit, the tension in the elastic wire unit will increase, resulting in an upward force. The upward force of the seat mechanism ensures that it is always vertical and ensures the stability of the wearer. For the standing scene, the left and right elastic wire units produce equal upward force on the seat unit. For the walking scene, the effect of each elastic wire unit on the seat unit will vary according to the supported legs. During the experiment, motion capture equipment and load cells were used to obtain six kinds of springs with different stiffness (580 N/m, 680 N/m, 970 N/m, 1317 N/m, 1734 N/m and 3327 N/m). The wearer can produce an upward support force of 9.41~26.18% of his own weight in standing posture and five different levels of spring stiffness. The exoskeleton provides an upward peak force ranging from 14.02 to 27.52% of the wearer’s weight when walking [34].

In 2019, Hebei University of Technology in China designed an assisted weight-bearing lower extremity exoskeleton (Figure 5g) consisting of a front branch and a rear branch, with variable stiffness spring energy storage elements. The principle of assisted load bearing is that one of the double-branched chains acts as the main transmission branch during walking, transferring the load from the human body to the ground. The experiment results show that when the wearer carries a load of 30 kg and 40 kg and walks 50 m, 100 m, 150 m, and 300 m, the total torque of the lower limb joints is reduced by 16%, and the metabolic energy consumption can be reduced by more than 10% [86,87,88].

In 2020, Beijing University of Aeronautics and Astronautics designed a passive lower limb exoskeleton with hip and knee joints (Figure 5h) that is used for assisted walking of patients with lower limb motor dysfunction. It consists of an energy storage unit composed of a tension spring and a rotating gear with a certain preload, with a mass of about 2.2 kg. The exoskeleton leg is suitable for people with a height of 1.60 m to 1.90 m, covering more than 90% of the corresponding adult men. During the experimental test, quantitative data such as EMG and metabolic cost were not used; only the subjects’ perceptual assistance was used to evaluate the gravity compensation performance of exoskeleton. The simulation analysis of different gait cycles, spring stiffness, preload, and limb mass shows that when the gait cycles are 3s, 5s, and 8s, the average hip joint torque is reduced by 53.2%, 79%, and 84.9%, respectively. The average knee joint torque decreased by 49.7%, 66.4%, and 69.8% respectively [89].

In 2021, Queen’s University of Canada developed a lightweight backpack exoskeleton for assisted walking and energy collection (Figure 5i) that is mainly composed of cables and rotating generators, with a mass of 1.059 kg. The two ends of the cable of the device are tied to the lower leg of the wearer and extend upward and then are connected to the input pulley. In each walking cycle, when the user’s knee extends forward to do positive work, the cable is released; at the moment of “braking” of the swinging leg before the foot falls to the ground, the cable is tightened, and the resistance from the generator provides a slight braking force to help the leg “brake”, also reducing the metabolic consumption of walking. At the same time, the cable drives the generator to rotate and convert the collected kinetic energy into a small amount of electric energy. The experimental results show that during the swing of the gait cycle, the exoskeleton reduces the metabolic cost of walking by 2.5 ± 0.8% for healthy male users and converts the removed energy into 0.25 ± 0.02 watts of power. At the same time, the gait parameters have little effect, realizing the breakthrough of negative cost of harvesting (COH) [90].

#### 3.2.2. Hip Assisted Exoskeleton

**Figure 6:**
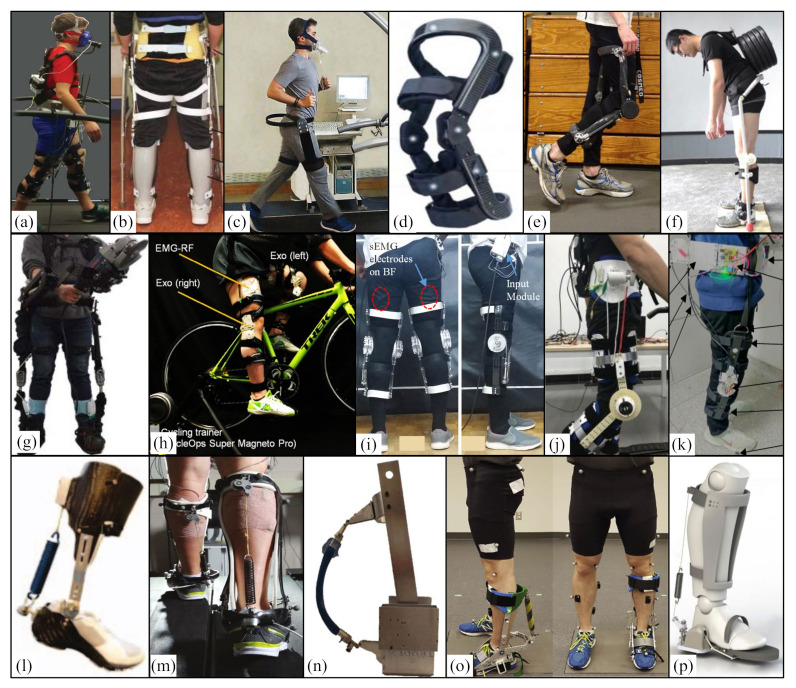
Single-joint passive lower limb assisted exoskeletosn. (**a**) Delft University of Technology, PHE [91]. (**b**) Tsinghua University, ES-EXO [93]. (**c**) Tehran University, UEFH [94]. (**d**) SLT, Levitation [95]. (**e**) MIT, Levitation [96]. (**f**) Chongqing University of Technology, PKAEXO [97]. (**g**) Hanyang University, M-ICR [98]. (**h**) Tohoku University, UKE [99]. (**i**) University of moletuwo, Sri Lanka, PPKE [100]. (**j**) South China University of Technology, UFLLE [101]. (**k**) Nanjing Southeast University, WBCAER [102]. (**l**) North Carolina State University, AAPE [103]. (**m**) Carnegie Mellon University, UE [104]. (**n**) University of Ottawa, UAE [105]. (**o**) University of Ottawa, PAE [106]. (**p**) Beijing Jiaotong University, PAFE [107].

In 2016, Delft University of Technology in the Netherlands designed a hip exoskeleton robot (Figure 6a) that is mainly composed of a hip device, leg device, and leaf spring, with a total weight of 4.15 kg. The hip device is a hip orthotic device with a steel structure. The leg device is divided into upper and lower parts that are connected and worn at the knee joint through a leaf spring. During the experiment, a healthy subject was tested on the treadmill at 1 m/s and 1.25 m/s to evaluate the performance of the passive hip exoskeleton, which verified the hypothesis that using the exoskeleton for walking can reduce energy consumption. During the experiment, the main work is as follows: (1) EMG signals were collected around the left hip joint to better understand which muscles are active in different gait stages; (2) the changes in walking metabolic cost were tested by obtaining the flow of oxygen and carbon dioxide; (3) for inverse kinematics and dynamics analysis, the coordinates of the lower limbs and the forces and moments on the treadmill were tracked. Compared with not wearing the exoskeleton, the metabolic cost increased by 25.9% and 22.9% at the two walking speeds. In the exoskeleton walking test, compared with the exoskeleton without the leaf spring, the metabolic cost increased by 11.8% and 0.5%. The metabolic energy consumption under the two walking speeds was nearly the same, indicating that the metabolic energy consumption of the wearer was reduced to a certain extent. Theoretical optimization showed that the metabolic energy consumed by the hip joint could be reduced by up to 60% without considering the metabolic increase caused by additional weight and gait changes compared with conventional gait [91].

In 2016, Tsinghua University in China designed an unpowered energy storage exoskeleton (ES-EXO) for the rehabilitation of patients with spinal cord injury (Figure 6b) that is composed of a bilateral handle shell, handle rod, knee lock, femoral shell, and femoral rod and two upward extending energy storage components. The device can provide specific walking assistance for patients with spinal cord injury by adjusting the energy storage element according to the characteristics of patients with spinal cord injury. The ground reaction force, EMG, and motion data were recorded during the experiment. The spring position and stiffness in the energy storage unit were optimized in the Anybody Modeling System simulation software. The optimization results showed that the hip flexion moment decreased by 37.2%, the abdominal muscle activity decreased, and the lumbar and back muscle activity increased slightly [92,93].

In 2018, Tehran University of Iran designed an unpowered exoskeleton (Figure 6c) that is realized by a bending leaf spring (BLS) and two frames, with a mass of 1.8 kg. The bending leaf springs of the hip joints on both sides of the wearer twist them in the swing stage to provide the wearer with passive hip extension torque and reduce metabolic energy consumption. The experimental results show that at the speed of 2.5 m/s, the wearer’s metabolic energy consumption decreased by 8 ± 1.5% without affecting the coronal movement of the wearer [94].

#### 3.2.3. Knee Assisted Exoskeleton

In 2012, Spring Loaded Technology in Canada launched a wearable bionic exoskeleton robot at the knee named the Levitation knee assisted exoskeleton (Figure 6d). When the knee is bent, the liquid spring in the exoskeleton device stores energy by compressing silicon fluid molecules. When the knee is extended, the exoskeleton releases energy to help the human body, with a mass of only 0.9 kg. The experimental results show that this knee-assist device can reduce the energy consumption of the wearer’s knee by up to 64% [95].

In 2013, MIT designed an elastic knee exoskeleton (Figure 6e), with a mass of 0.71 kg. The exoskeleton consists of a composite leaf spring parallel to the leg and connected to the clutch at its midpoint, so that the spring may not provide stiffness during the swing phase. However, locking before standing provides stiffness when standing. Six men were used to run on a treadmill at a speed of 3.5 m/s. The kinematics and dynamics of joints, EMG, and metabolic needs were measured. The experimental results show that the reactions of leisure runners and competitive long-distance runners are different. Their total knee torque increases due to wearing equipment, and the increase in knee mass will increase the stiffness in the legs. There are no changes in leg stiffness and knee stiffness before and after exoskeleton wearing [96].

In 2017, Chongqing University of Technology in China designed a passive knee assist exoskeleton design for weight-bearing climbing (Figure 6f) that mainly uses Teflon rope, a pulley, and a compression spring to form an energy storage unit. The shape of the pulley for winding is designed to be eccentric, and the auxiliary torque changes nonlinearly with the knee flexion angle. Through the mechanical modeling of the eccentric pulley, the auxiliary torque of the device is predicted and compared with the actual experimental data. Experiments show that the exoskeleton can bear a maximum load of 70 kg, which can reduce the maximum average force of the knee extensor by 21% [97].

In 2018, Hanyang University of Korea proposed a negative heavy-duty lower limb knee exoskeleton robot (Figure 6g). It is a four-bar linkage with cam follower and movable instantaneous rotation center (M-ICR), including a support frame connecting the thigh and calf. The mechanism realizes two functions at the same time: (1) during the extension of the knee joint, it performs mechanical locking and transfers the load (20 kg in this study) to the ground; (2) while the knee joint is in the swing phase, it releases the lock and moves with the wearer. The subjects carried 0 kg, 10 kg, and 20 kg of external load and walked on the treadmill at the speed of 2 km/h, and the oxygen consumption and vertical ground reaction force were tested. The test results show that: (1) only when 20 kg load is applied did the oxygen consumption decrease slightly; (2) the vertical ground reaction force increased by 19% when there was no load and decreased by 28% and 36% when the loads were 10 kg and 20 kg, respectively. When the wearer used the grinder (5.5 kg), the ground vertical reaction force decreased by 10%, and the oxygen consumption decreased after 90 s compared with the case when the subject did not wear the exoskeleton [98].

In 2018, Tohoku University of Japan designed an unpowered knee exoskeleton for riding assistance (Figure 6h) that is composed of a cross four-bar mechanism and an embedded torsion spring, with a total mass of 1.07 kg. During knee flexion, the torsion spring compresses and collects energy and releases the stored energy during knee extension. In the experiment, the wheel acceleration system is designed to ensure constant power cycle at constant speed and constant torque. The rear wheel starts to rotate to the target speed of 30 km/h, corresponding to the riding power with 200 W and 225 W training. The experimental verification results show that compared with the riding test without the exoskeleton, the activity of quadriceps femoris decreases, and the average EMG density during cycling increases with the increase in exercise intensity [99].

In 2018, Moletuwo University of Sri Lanka proposed a knee exoskeleton with a passive dynamic mechanism (Figure 6i) that provides dynamic assistance to the knee when squatting and lifting objects. The shell adopts an acrylic plate, and the interior is composed of energy capture/storage springs parallel to the rod and a steel wire rope, pulley disc, small return spring, and pin, with a total mass of 1.8 kg. When the knee flexion angle of the wearer exceeds 60° in the squatting stage, the energy of the knee joint is stored, and the energy is released for assistance in the standing stage. The performance of key muscles of knee joint was evaluated by surface electromyography (sEMG), which verified the effectiveness of the system. The experimental data showed that the peak root mean square of the EMG signal of the knee extensor muscle decreased by 30~40% when subjects squatted with the exoskeleton [100].

In 2019, South China University of Technology developed a flexible lower limb exoskeleton for assisted walking and energy collection (Figure 6j). Half of the exoskeleton includes hip and knee auxiliary mechanisms that are connected through inelastic cables with a total mass of 1.7 kg. When worn on lower limbs, the exoskeleton can partially replace the function of lower limb muscles. It can remove kinetic energy when the lower limbs decelerate and assist in accelerating movement, so as to reduce the biomechanical power consumption during walking. In addition to assisted walking, the generator in the exoskeleton acts as a damping element that can collect kinetic energy and power wearable electronic devices. The experiment shows that when the walking speed is 4.5 km/h, the lower limb exoskeleton can reduce the metabolic cost by 3.12 ± 2.11%, and when the walking speed is 5.1 km/h, the maximum electric energy generated is 6.47W [101].

In 2022, Nanjing Southeast University proposed a knee-assisted soft exoskeleton with energy collection ability (Figure 6k). Instead of using a separate device for power assistance and energy collection, the exoskeleton uses a single electromagnetic unit to control the relay switch based on the operation mode of the user’s gait cycle. The experimental results showed that the thigh muscle activity decreased by an average of 7.91%, and the maximum power generated during downstairs exercise was 3.2 W [102].

#### 3.2.4. Ankle Assisted Exoskeleton

In 2011, North Carolina State University designed a portable elastic ankle exoskeleton (Figure 6l) to assist ankle propulsion through energy storage and release. It is inspired by the passive elastic mechanism of the human triceps brachii-Achilles tendon complex during walking, with a mass of 0.57 kg. A new type of “intelligent clutch” is developed that can start and release a parallel spring only according to the motion state of the ankle joint. A linear tension spring (stiffness 23.4 N/mm, torque arm 126 mm, maximum torque 109 N/mm, elastic energy 20.7 J) is used to make the energy storage spring stretch and contract regularly to store and release energy according to the ankle angle. It can help with walking and reduce human metabolic energy consumption. Considering the normal start time of soleus and gastrocnemius muscle activation, the key points of walking stride were determined to engage (10% stride cycle) and disengage (60% stride) clutches. The test of the device showed that it can easily support a user with a weight of 75 kg, and the safety factor is 2.5 [103].

In 2015, Collins of Carnegie Mellon University designed an unpowered ankle exoskeleton (Figure 6m) that can reduce the metabolic rate of human walking. It is composed of a lightweight elastic device. This device uses a mechanical clutch to control the spring to help complete the function of the calf muscle and the Achilles tendon. The weight of the exoskeleton clutch is 0.057 kg, and the mass of a single exoskeleton is 0.408~0.503 kg. During the experiment, nine participants were tested. Each leg wore the exoskeleton and walked on a treadmill at 1.25 m/s. The results showed that: (1) the ankle torque produced by the calf muscle was reduced, mainly the activation of the calf soleus muscle; (2) the joint angle changed little under different conditions; (3) when springs with different stiffness were used, the metabolism first decreased and then increased with increases in the spring stiffness; (4) when the elastic modulus was 180 Nm/rad, the metabolic cost of walking was reduced to 2.67 ± 0.14 W/kg, while that of normal walking was 2.88 ± 0.10 W/kg, which was reduced by 7.2 ± 2.6%; (5) the metabolic energy (net metabolic rate) used for walking was 1.47 ± 0.10 W/kg, which is equivalent to an ordinary person carrying a 4 kg backpack [104].

In 2018, American HeroWear company, Yandell et al. optimized the structure, appearance, and wearability of the unpowered lower limb ankle assisted exoskeleton designed by Collins et al. in 2015. It integrates the foot clutch, soft conformal calf interface, and ankle auxiliary spring, which works in parallel with the user’s calf muscles. The device simplifies the clutch device and eliminates the use of the additional mechanical structure. The device similar to the insole is connected in series with the spring using the pressure of gravity on the shoes when people walk. The innovative design of the energy storage clutch not only makes the power-assisted device easier to carry, lighter in weight, and higher in space utilization, it also reduces the energy loss during energy transmission. It effectively improves the energy utilization of the system and reduces the metabolic energy of the calf muscles. This is the world’s first lower limb power-assisted exoskeleton without a power source that uses plantar flexors to assist, and it is also the first power-assisted device that can be completely hidden under daily wear. It highlights the development potential of lower limb assisted exoskeletons in personal life and society [108].

In 2018, the University of Ottawa, Canada, developed an unpowered ankle exoskeleton for assisted walking (Figure 6n). The spring characteristics of an inflatable and sealed pneumatic artificial muscle are used, and the mass is 1.35 kg. The pressure of the pneumatic artificial muscle is 206Kpa, the diameter is 1.3 cm, the compression length is 19.2 cm, and its stiffness varies from 3.5 to 6.5 kN/m. The device can release energy when the ankle is in the swing phase, and the energy can be stored at the toe of the foot. In order to verify that the exoskeleton device released similar torque to the theoretical ankle joint, a test bed was made for the experiment. The experimental results show that for a wearer of 80 kg, the maximum torque of the exoskeleton can reach 115 N/m, and the maximum torque without the exoskeleton in the normal gait is 128 N/m [105,109].

In 2019, the University of Ottawa in Canada developed a unilateral ankle exoskeleton (Figure 6o) that uses a pneumatic artificial muscle as a nonlinear elastic element with a mass of 1.51 kg. The device integrates a novel clutch mechanism design that can engage and separate the spring elements of the ankle during walking, so as not to hinder the ankle movement in the swing stage. Experimental verification shows that wearing the exoskeleton can give full play to its function within the natural range of ankle and produce an ankle moment equivalent to at least 25% of the natural ankle moment during normal walking. The EMG signal of the gastrocnemius muscle is reduced, but the use of the high-pressure pneumatic muscle will reduce the motion range of the ankle. The anterior tibial band of the tibial muscle decreased, and the anterior tibial band of the tibial muscle increased. This may be due to the fact that the lower limb muscles compensate for the abnormal feeling when walking with exoskeleton without prior training [106].

In 2019, Beijing Jiaotong University developed a spring-driven ankle exoskeleton (Figure 6p). It includes a sensing part and an executive part, and the executive part includes a pulley, pre-tension spring, and rope. The structure of the device is similar to the tendon muscle unit, and the spring is suspended in parallel behind the calf muscle. When the human body swings, it stores energy (clutch state) and converts energy into strain energy during back flexion (the clutch switch will be released). In a certain range, the higher the spring stiffness, the more energy can be recovered. After the simulation analysis using OpenSim software and five cases, the strength and energy of the soleus and gastrocnemius were obtained. It was decided to choose a spring with a stiffness of 25 N/mm, a device that reduces the wearer’s metabolic energy consumption [107].

Similarly, exoskeletons designed in many countries are used to decrease the metabolic energy consumption of human walking. For example, an ankle exoskeleton designed by the Ghent University in Belgium can reduce the metabolic cost by 0.186 ± 0.06 w/kg [35], but the exoskeletons cannot walk.

To sum up, the unpowered exoskeleton robot does not need an additional drive system and energy supply system, and it can achieve the same performance as the powered exoskeleton robot. With the growing aging population and movement disorders, people pay more and more attention to the need for rehabilitation assistance and the functional loss caused by high-intensity labor. This gives the unpowered lower limb exoskeleton very broad application prospects. However, at present, the unpowered lower limb exoskeleton robot has shortcomings in human-computer coordination, control effects, and auxiliary effects. In addition, the single-joint unpowered exoskeleton robot with lighter weight, smaller volume, and lower cost will be more and more popular and even promote the development of multi-joint exoskeleton robots.

### 3.3. Quasi-Passive Lower Limb Exoskeleton

In the past decade, there has been a steady increase in research and development on active and passive exoskeletons for the prevention of work-related injuries. In recent years, new quasi-passive exoskeleton designs are on the rise. They use passive viscoelastic elements (such as springs and dampers) to support the user, while small actuators are only used to change the level of support or to disengage the passive elements. In order to maximize the use of passive viscoelastic elements, the control of such devices, especially the algorithm of predicting user movement, remains to be explored to a great extent.

#### 3.3.1. Multi-Joint Assisted Exoskeleton

**Figure 7:**
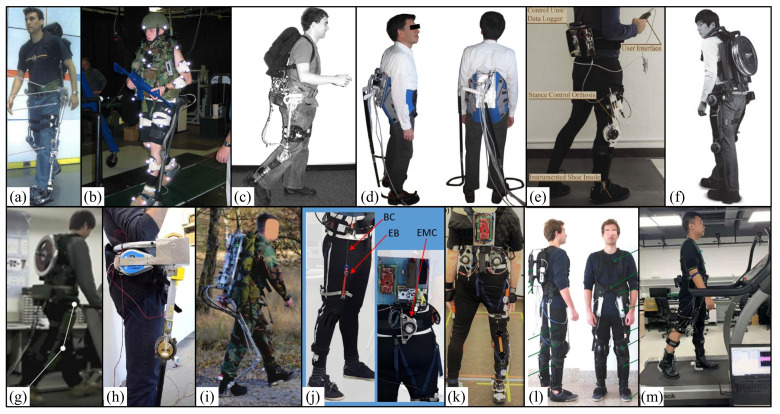
Multi-joint lower limb assisted exoskeletons. (**a**) MIT, QPPLEALCW [110]. (**b**) US Army Natick soldier Center, LBELCAD [112]. (**c**) MIT, QPLELCA [3]. (**d**) University of Montpellier, Moonwalker [113]. (**e**) Yale University, QPCSCKAFO [114]. (**f**) Hanyang University, HEXAR, Seoul, Korea [116]. (**g**) Korea Atomic Energy Research Institute, UEEWLCA [118]. (**h**) Tokyo University of agricultural technology, QPLLEPBWS [119]. (**i**) Netherlands applied scientific research organization, Exobuddy [120]. (**j**) Italian Institute of Technology, SWDLLA [121]. (**k**) Italian Institute of Technology, SALLE [122]. (**l**) Italian Institute of Technology, SALLE [123]. (**m**) Shenzhen Institute of Advanced Technology, Chinese Academy of Sciences [124].

In 2005, one study was completed under Defense Advanced Research Projects Agency (DARPA) contract #NBCHC040122: “Leg orthosis for sports endurance amplification”. MIT introduces a quasi-passive leg exoskeleton (Figure 7a) that is designed to help humans carry a 75-pound payload. To make the exoskeleton more efficient, passive hip and ankle springs are used to store and release energy throughout the gait cycle. In order to reduce the strength of knee muscles, a variable damper is installed at the knee to support weight throughout the early standing process. During the experiment, a participant measured his oxygen consumption while walking on a horizontal plane at a selected speed. The experimental results show that compared with the 75-pound backpack without the exoskeleton, the quasi-passive exoskeleton with the 75-pound backpack increased the metabolic cost by 39%. When the variable damper knee was replaced by a simple pin joint, the metabolic cost relative to no auxiliary load decreased to 34%, indicating that the damping advantage of the damper knee cannot compensate for its increased mass [110].

In 2006, MIT tried to understand how leg muscles and tendons work mechanically during walking, so as to stimulate the design of efficient robot legs. Their device includes spring, clutch, and variable damping elements that can capture the main mechanical behavior of the human knee and ankle when walking on horizontal ground at an independently selected speed. Ken Endo et al. [111] proposed a simple leg model that captures the general characteristics of the human leg musculoskeletal structure. By changing the spring constant, damping level, and clutch engagement time, an optimization scheme was used to minimize the error between the model joint behavior and the biological joint mechanics [111].

In 2006, the U.S. Army Natick Soldier Center designed a negative heavy lower limb exoskeleton (Natick exoskeleton) (Figure 7b). The effects of the device on oxygen consumption and kinematics during weight-bearing walking were studied. Ten Army soldiers participated in the study. During walking at a horizontal degree of 4.83 km/h, oxygen consumption (VO2) and gait biomechanics were measured under three load configurations. This study showed that load transport with the Natick exoskeleton device was associated with a 60% increase in metabolic COT under all three test load conditions (20 kg, 40 kg, 55 kg) [112].

In 2007, MIT designed a quasi-passive leg exoskeleton for weight-bearing enhancement (Figure 7c). There are two single joint springs at the hip and ankle, and a variable damping device at the knee constitutes the core energy storage structure, with a total mass of 11.7 kg. During the experiment, a male subject participated in the study and walked at 0.92 m/s in different experimental environments. The study found that the quasi-passive exoskeleton increased the metabolic rate by 10% compared with a standard weight-bearing backpack. The metabolic data showed that the zero-impedance exoskeleton increased by 23% compared with the weight-bearing backpack and 12% compared with the quasi-passive exoskeleton. This means that the use of the quasi-passive exoskeletons reduced metabolism compared with zero-impedance exoskeletons. This illustrates the benefits of spring and variable damping mechanisms for ankles, knees, and hips [3].

Also in 2010, the lunar Walker of the University of Montpellier in France was made (Figure 7d), which is a lower limb exoskeleton that can bear part of the user’s weight. The exoskeleton can be used for rehabilitation to help people with weak legs or broken legs walk. It can also be used as an auxiliary device to help people carry heavy objects. Its main feature is that it is provided with the force to support the weight by the power balancer. An actuator is also required, but only to move the force to the same side as the leg standing posture. Therefore, the lunar rover needs very low energy to work on flat terrain. The motor can provide part of the energy to climb stairs or slopes. This method helps to improve the energy autonomy of the lower limb exoskeleton [113].

In 2013, with the support of the US Defense Medical Research and Development Program (grant# W81XWH-11-2-0054), Yale University proposed a new type of quasi-passive attitude control knee-ankle-foot orthosis (Figure 7e). When standing, the unaffected knee behaves close to a linear torsion spring. Kamran Shamaei et al. Introduced the design of a friction-based locking mechanism and a control algorithm that makes the spring mesh parallel to the knee in the standing state and separates it in the gait in the swing phase. The device is quasi-passive and uses a small actuator to lock and unlock the spring module, but the device does not need to be driven and requires little power, calculation, or control during operation [114]. After that, preliminary tests were conducted on three uninjured subjects. The results show that compared with the rigid support provided by the advanced commercial posture control orthosis, the knee-compliant support provided by the orthosis can improve the gait speed and make the lower limbs move more naturally [115].

In 2014, the Hanyang Exoskeleton Assisted Robot (HEXAR) [116] was designed by Hanyang University in Seoul, Korea (Figure 7f). It is used to assist individuals with weight bearing and is an underdriven wearable exoskeleton system. The exoskeleton consists of a central torso bundle and hip, knee, and ankle joints with a total of 15 degrees of freedom. The hip joint uses a constant force mechanism to carry the weight and load of the upper exoskeleton system. The ankle joint carries the total weight of the exoskeletal system and powers the walking by its own elastic potential energy. The wearer can walk at a speed of 6 km/h under a load of 35 kg.

In 2015, based on passive walking theory, a quasi-passive energy-saving assisted lower limb exoskeleton was designed at Southeast University. A two-dimensional passive bipedal walking model is described, and an energy-saving method for powering the model on flat ground is proposed. An energy-efficient lower limb exoskeleton is proposed based on a realistic two-dimensional model of passive bipedal walking, and a control strategy combining hip joint rotational drive and push-off compensated drive is used to construct an energy-efficient lower limb exoskeleton [117].

In 2015, Seungnam Yu et al. of the Korean Atomic Energy Research Institute proposed and developed an underactuated exoskeleton (Figure 7g) to support external loads and partially assist leg movements, including horizontal walking and rising slopes and stairs, that need to generate positive energy. The strategy of active and passive joint combination and a quasi-passive mechanism are realized on the underactuated exoskeleton to help vertical weight support and gait propulsion, while minimizing the obstacles to the free movement of the wearer. Through the exoskeleton system experiment and analyzing the EMG data of the fremortus and gastrocnemius muscles when walking and climbing stairs, the considered performance is verified. The developed underactuated exoskeleton can help healthy people carry out weight bearing and promote effective lifting through structural weight support, leg swing, and lifting motion assistance through motorized knee joints. This active joint minimization method is particularly useful in field applications that require independent power sources such as batteries [118].

**Figure 8:**
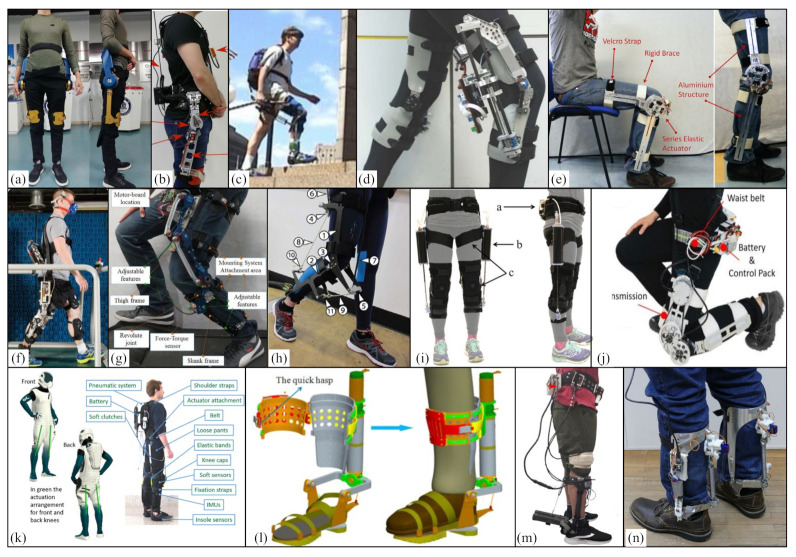
Single-joint quasi passive lower limb exoskeletons. (**a**) Chinese Academy of Science (CAS) [125]. (**b**) Slovenia, CQPSE [126]. (**c**) University of Michigan, RoboKnee [127]. (**d**) Carlton University, QPKEAR [128]. (**e**) Italian Institute of Technology, ACCKE [130]. (**f**) Yale University, QPKEGE [132]. (**g**) Italian Institute of Technology, iT-Knee [133]. (**h**) Carnegie Mellon University, LTTCKE [134]. (**i**) Harvard University, QPKEADD [135]. (**j**) City University of New York, LBKE [5]. (**k**) Italian Institute of Technology, QPRESA [136]. (**l**) Harbin Institute of Technology, QPAFWRO [137]. (**m**) University of Texas, SATQPAE [138]. (**n**) Ljubljana, Slovenia, CAAE [139].

In 2016, Andrea Collo et al. at the Tokyo University of Agricultural Technology in Japan proposed a strong, universal, and economical exoskeleton (Figure 8h) that can combine partial weight support and the high portability provided by crutches. The device relies on the use of passive components and only two compact drivers with a mass of 9 kg. Manufacturing a conceptual prototype allows a qualitative evaluation of the working principle of the system. In the case of wearing the equipment, horizontal walking, squatting, sitting up, and sitting up movements were successfully carried out. The experimental results show that the weight of users wearing seat belts is reduced by about 30 kg [119].

In 2018, W. van Dijk et al. of the Netherlands applied scientific research organization (TNO) and introduced an exoskeleton (Figure 7i) with a mass of 21.8 kg. The Exobuddy transfers part of the load directly to the ground. The Exobuddy mechanism is quasi-passive, thus eliminating the need for large energy sources associated with active exoskeletons. During the experiment, 9 subjects were recruited from the Dutch marine corps to participate in indoor and outdoor experiments with and without exoskeletons. The outdoor environment was walking on a 1.6 km asphalt road and a 3.2 km dirt road (sand) and partially downhill and uphill. The indoor environment was tested on the treadmill at speeds of 4.5–5.3 km/h and the maximum bicycle test. The experimental results show that 29.4% and 29.9% of the loads were externally supported. After heel impact, an average part (about 30%) is transferred to the ground, with a maximum of about 53% [120].

In 2018, in work that was funded by the EU horizon 2020 Research and Innovation Framework Program (Grant Agreement No. 688175), Tommaso Poliero et al. of the Italian Institute of Technology (Figure 7j) proposed a soft modular energy-saving lower limb exoskeleton design with a mass of 1.94 kg. It is based on energy efficiency analysis and optimization. The novelty of this work lies in the integration of the quasi-passive drive-in soft exoskeleton. The goal is to help subjects with low to moderate activity disorders reduce mechanical energy requirements by 10% to 30% and improve gait (stability, fatigue, etc.) in daily tasks. Assistance refers to unilateral or bilateral configuration of the hips, knees, and ankles. The first modular single joint prototype is described, developed, and evaluated after stroke patient gait. The comparison between validation and simulation shows similar behavior, and the energy is reduced by 7.8%. The overall strength measurement help provided by the exoskeleton in the user’s hip is 9.3% [121].

In 2019, Christian Di Natali et al. of the Italian Institute of Technology proposed a soft modular lower limb exoskeleton design (Figure 7k) to improve human flexibility, help with independence, and improve the quality of life, with a mass of 4.6 kg. The novelty of this work is the integration of quasi-passive elements in the soft exoskeleton. The exoskeleton provides mechanical assistance to subjects with low dyskinesia, reducing energy requirements by 10% to 20%. Different control strategies based on gait segmentation and driving elements were studied. The first unilateral hip knee prototype was described, developed, and evaluated for its performance in post-stroke patients walking straight. In this study, the energy mode of human exoskeleton was analyzed using task-based bioelectricity. In terms of power, the hip drive was 10.9 ± 2.2% and knee drive was 9.3 ± 3.5%. This control strategy improved gait and posture patterns by increasing the joint angle and foot clearance at specific stages of the walking cycle [122].

In 2020, Christian Di Natali et al. of the Italian Institute of Technology designed a bionic soft and modular exoskeleton assisted by lower limbs based on quasi-passive pneumatic drive (Figure 7l). It is mainly composed of a pressure sensor, solenoid valve, and vacuum generator, with a total mass of 0.8 kg. The passive belt is driven by a traditional adjusting element. During the experiment, participants were evaluated during a 10 min walking task on a treadmill, and the parameters of the exoskeleton and motion tracking were recorded during this period. The second test was conducted along a 10 m straight path in which the kinematics of the limbs and ground reaction forces were measured to analyze the effectiveness of the transmission assistance. The experimental results show that the total power ratio between the exoskeleton and the auxiliary joint was 26.6% for the hip, 9.3% for the knee, and 12.6% for ankle. The maximum power released by each joint was 113.6% for the hip, 93.2% for the knee, and 150.8% for the ankle [123].

In 2021, Shenzhen Institute of Advanced Technology, Chinese Academy of Sciences developed a light soft exoskeleton (Figure 7m). During the gait cycle, the automatic winding device uses only one motor to assist the hip plantar flexion of both legs at different stages. The weight of the whole system is only 2.24 kg. Six subjects were tested with and without the exoskeleton. Compared with the case without the exoskeleton, the muscle fatigue in the rectus femoris, lateral femoris, gastrocnemius, and soleus decreased by 14.69%, 6.66%, 17.71%, and 8.15% respectively [124].

#### 3.3.2. Hip-Assisted Exoskeleton

In 2019, the Chinese Academy of Science (CAS) developed a wearable lumbar exoskeleton (Figure 8a). The exoskeleton is fixed on the wearer’s body by four belts. It can be put on in only 30 s and weighs only 5 kg. With and without the exoskeleton, six different objects (0, 5, 10, 15, 20, 25 kg) were lifted. The exoskeleton significantly reduced back muscle activity. The average comprehensive EMG of lumbar erector spinae (LES), thoracic erector spinae (TES), and latissimus dorsi (LD) decreased by 34.0%, 33.9%, and 24.1% respectively [125].

In 2020, Marko Jamšek et al. from Slovenia proposed a control scheme based on a combination of a Gaussian mixture model (GMM) and a state machine operator. The user’s behavior was identified and classified as quickly as possible in order to provide immediate operational output towards a quasi-passive spine exoskeleton (Figure 8b). In eliminating cross-validation, the total improvement in the overall accuracy of the supported users was 86.72 ± 0.86% (mean ± standard deviation), with sensitivities and specificities of 97.46 ± 2.09% and 83.15 ± 0.85%, respectively. The findings of this research suggest that the method is a highly promising instrument for the control of quasi-passive spinal exoskeletons [126].

#### 3.3.3. Knee-Assisted Exoskeleton

In 2004, a robot knee joint was designed by Jerry et al. of the University of Michigan (Figure 8c). It is a single degree of freedom exoskeleton that can achieve high transparency. The user’s intention is determined by the knee angle and the ground reaction force. In order to relax the user’s quadriceps, torque is applied to the knee joint. Low impedance is achieved using a series elastic actuator. The RoboKnee allows the wearer to climb stairs and bend the knees while carrying heavy objects. As the user maintains control over when and where to walk, and provides balance and control, the device provides most of the energy required to work against gravity [127].

In 2008, Aaron M et al. of Carlton University in the United States developed a quasi-passive knee exoskeleton for assisted running (Figure 8d). The core power unit of its assisted running function is composed of spring, clutch, and variable damping components, with a total mass of 11.7 kg. The device consists of a knee support in which an electric mechanism actively places and removes the spring parallel to the knee. The current design saves 7.8 J positive mechanical energy and 6.1 J negative mechanical energy of the knee, or 19% and 11%, respectively [128].

In 2012, Grant A. Elliott [129] of Massachusetts Institute of Technology proposed a knee exoskeleton, including a leaf spring parallel to the knee and a clutch that uses this spring only when standing. The design of a custom interference clutch has an unacceptable 1.6 kg even for the lightest commercial equipment that provides sufficient holding torque. The effect of parallel elasticity on human running was studied.

In 2014, Nikos Karavas et al. of the Italian Institute of Technology proposed an auxiliary control scheme of knee exoskeleton equipment based on distal impedance (Figure 8e). The controller captures the user’s intention to generate task-related auxiliary torque through the exoskeleton at different stages of normal activity. To this end, a detailed human knee musculoskeletal model is developed and calibrated through experiments to best match the user’s kinematic and dynamic behavior. In addition, the experimental results of standing and sitting tasks are demonstrated, and the performance of the controller is further studied. The results show that the flexible knee exoskeleton, combined with the proposed remote impedance controller, can effectively generate auxiliary actions that are voluntarily and intuitively controlled by the user’s muscle activity [130].

In 2014, this work was supported by the U.S. Army Natick Soldier Research, Development and Engineering Center (Contract No. #W911NF-07-D-0001). Yale University Kamran Shamaei et al. [131] studied the auxiliary effect provided by a quasi-passive knee exoskeleton (Figure 8f). The knee exoskeleton is composed of motors, worm gear sets, and springs assembled on the thigh segment of the exoskeleton. The exoskeleton mass of the left leg and right leg is 2.63 kg and 2.45 kg, respectively. Using exoskeletons in a series of experiments with seven participants, it was found that increasing the exoskeletons can reduce muscle strength and work, so as to reduce the metabolic cost of walking. At the same time, Kamran Shamaei et al. [132], in studying the biomechanical behavior of knee joint when interacting with external applied impedance, found that the quasi-passive assistance of exoskeleton can help reduce the torque distribution of human knee joint in the sagittal plane.

In 2016, the Italian Institute of technology Lorenzo saccares et al. Proposed a new exoskeleton system for knee rehabilitation/dynamic enhancement (Figure 8g). The device is an underactuated 6-DOF self-aligning knee exoskeleton. It provides pure auxiliary torque for the flexion and extension of the knee, while all other movements of the knee are passive. Therefore, the device has no restrictions on the user’s actions. The iT-Knee can automatically adjust the misalignment between the rotating shaft and the knee joint, which is suitable for users of different sizes. Therefore, not only will there be no unnecessary force/torque between the user’s leg and the exoskeleton attachment but also the installation and installation time required by the iT-Knee are reduced. The torque control mode of the iT-Knee during normal walking also achieved encouraging results with the torque reference set to zero [133].

In 2017, Kirby et al. of Carnegie Mellon University designed and manufactured a tethered knee exoskeleton (Figure 8h) with a robust lightweight frame and comfortable four-point contact in the leg. The device has a flexible structure and can measure the joint angle and apply torque, and the mass is 0.76 kg. The exoskeleton is driven by two outboard motors. When the maximum load was 62.2 Nm, the measurement accuracy of torque was tested, and the root mean square error was 0.8 Nm. The bandwidth was phase limited to 45 Hz when measured on a rigid test bench and 23 Hz when measured on a human leg. Peak expansion torque of 50 Nm was achieved on the bandwidth test. The torque tracking was measured by walking on a treadmill at 1.25 m/s with a peak bending torque of 30 Nm. The root mean square of torque tracking error above 100 steps is 0.91 Nm [134].

In 2017, Emily Rogers et al. of Harvard University designed a quasi-passive knee joint pneumatic exoskeleton assisted during descent (Figure 8i) that aims to reduce the muscle activity of the knee extensor when walking on a negative slope. The device consists of an air spring that can be engaged and separated by a solenoid valve. When used, the air spring resists knee flexion. The preliminary evaluation of the device was performed with a healthy subject. On the test, the EMG activity of the rectus femoris decreased by 15%, while the EMG activity of medial femoral muscle increased by 8% [135].

In 2018, Wang et al. of City University of New York proposed a physically worn knee exoskeleton (Figure 8j) to overcome discomfort. The new wearable structure was analyzed and optimized and compared with the traditional method. The auxiliary control scheme is based on force control. Compared with the exoskeleton under position control, it has significant advantages of human-computer interaction. The kinematics simulation results show that the dislocation rate between robot joint and knee joint decreased by 74% at the maximum flexion of the knee joint. In the experiment, the exoskeleton showed a low resistance torque of 1.03 Nm root mean square (RMS) in unpowered mode. Torque control experiments on three subjects showed that the torque tracking error was 0.31 Nm rms [5].

In 2021, Christian et al. of the Italian Institute of Technology proposed a portable proprioceptive device for quasi-passive exosuit muscle training (Figure 8k). In daily activities, the exosuit promotes continuous exercise by resisting the user’s movement. The effectiveness of the exosuit was evaluated by analyzing the effect of resistance on muscle endurance on a land walking task. The experimental evaluation of the biceps femoris and lateral femoris showed that the average increase of muscle activation was about 97.8% in five 3-min walking tasks at 3 km/h. Power frequency analysis showed that muscle fatigue increased and the median frequency of EMG decreased by about 15.4% [136].

#### 3.3.4. Ankle-Assisted Exoskeleton

In 2015, Chao Zhang et al. of the Harbin Institute of Technology in China designed a quasi-passive 3-DOF ankle-foot wearable orthosis (Figure 8l). A combination of electromagnetic clutch, ball screw, and wire were used for the mechanical transmission. In the initial support phase, the solenoid clutch is dissociated and the spring is forced to store energy. During the later stages of support, the solenoid clutch becomes active, and the necessary ankle moment is supplied by the spring and motor. By combining passive energy storage with additional power supplementation, ankle trajectories and dynamics similar to natural gait were generated. In functional terms, the orthosis is vibration-damping and low-energy. Owing to its comfortable, light and human-like construction, the orthosis can be utilized in medical facilities for rehabilitation training or as a daily walking aid for surgical patients [137].

In 2020, Saurav Kumar et al. of the University of Texas developed a quasi-passive ankle exoskeleton (Figure 8l) that has a self-adjusting variable stiffness mechanism with a mass of 1.75 kg. Because the relationship between muscle strength and optimal spring stiffness under different walking speeds is unknown a priori, the model free discrete-time extreme value optimization control (ESC) algorithm is used to optimize the spring stiffness in real time. Experiments on a healthy subject show that ESC will automatically adjust the torsional stiffness of the ankle with changes in the user’s walking speed. During slow walking, the average RMS EMG readings of the tibialis anterior muscle and the soleus muscle decreased by 26.48% and 7.42%, respectively [138].

In 2020, the Slovenian state Miha Dezman et al. [139] improved the original passive ankle exoskeleton with an active clutch to make it quasi passive, with a mass of 1.090 kg (Figure 8m). The purpose was to alleviate the problems of clutch engagement and user physiological changes. To evaluate exoskeleton and clutch operation, seven participants tried to walk with the exoskeleton. Qualitative user feedback was collected focusing on equipment comfort, user perception of the exoskeleton’s effects, and the stability of the clutch operation, as well as quantitative data on the clutch operation during walking on a flat surface. The results show improved and more reliable exoskeleton clutch operation, which is also reflected in qualitative user feedback.

People invented shoes to provide users with comfortable rubber soles, and human action ability has been slightly improved. Different from shoes, at present, quasi-passive exoskeletons of lower limbs uss variable stiffness springs to store and recover energy to improve mobility. Among them, the maximum kinetic energy accumulated by human beings has nothing to do with limb deflection and the ability of limbs to produce strength. This is achieved by increasing the variable stiffness of the human body, and the exoskeleton does not provide mechanical work. The theoretical advantages provided by this new enhancement method are that it can be used for high-demand tasks and that humans can benefit from sports that improve speed and reduce energy cost [140].

To sum up, in order to make up for the shortcomings of unpowered exoskeleton in man-machine coordination, control effect, auxiliary effects, etc., and based on the unpowered exoskeleton, the quasi-passive exoskeleton introduces quasi-passive components with little power demand but still needs a power supply to operate the electronic control system, clutch or variable shock absorber. Multi-joint assist is mainly used for weight bearing, assisted walking, and knee-ankle-foot orthoses. In the aspect of single-joint assistance, there is much research on knee joint assistance, including weight bearing, going up and down stairs, carrying heavy objects, and assisted walking. Ankle assist is mainly used for ankle foot orthoses and assisted walking. The man-machine matching and control system accuracy of the quasi-passive device is still a problem to be solved. In the future, researchers should strengthen the exploration of human body mysteries and abandon the dynamic system. Only with the help of elastic elements and support components can the daily needs be met of all kinds of people to realize true man-machine integration.

## 4. Discussion

Table 2, Table 3, Table 4, Table 5, Table 6 and Table 7 summarize the different types of power-assisted exoskeletons. In recent years, the research on assisted exoskeletons has been increasing, but there are still many difficulties to be solved before a fully functional exoskeleton is developed. According to human biomechanics and energy conservation theory, a booster exoskeleton can be divided into passive or active. 

In addition, according to different working principles (different forms of energy storage elements), assisted exoskeletons were analyzed and discussed. We summarized the five main factors: quality, assistive effect, size, weight, and mounting. The capabilities of assistive exoskeletons, their disturbances to the body, and their effects on metabolism were assessed. The advantages and disadvantages of assisted exoskeletons are shown in Table 8.

### 4.1. Weight and Performance

**Figure 9:**
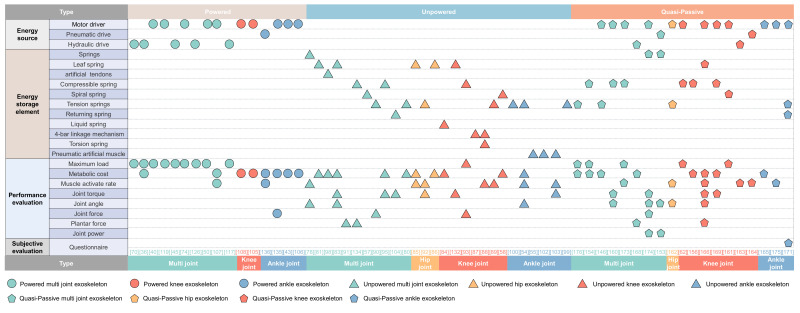
Classification of wearable lower limb assist exoskeleton.

**Figure 10:**
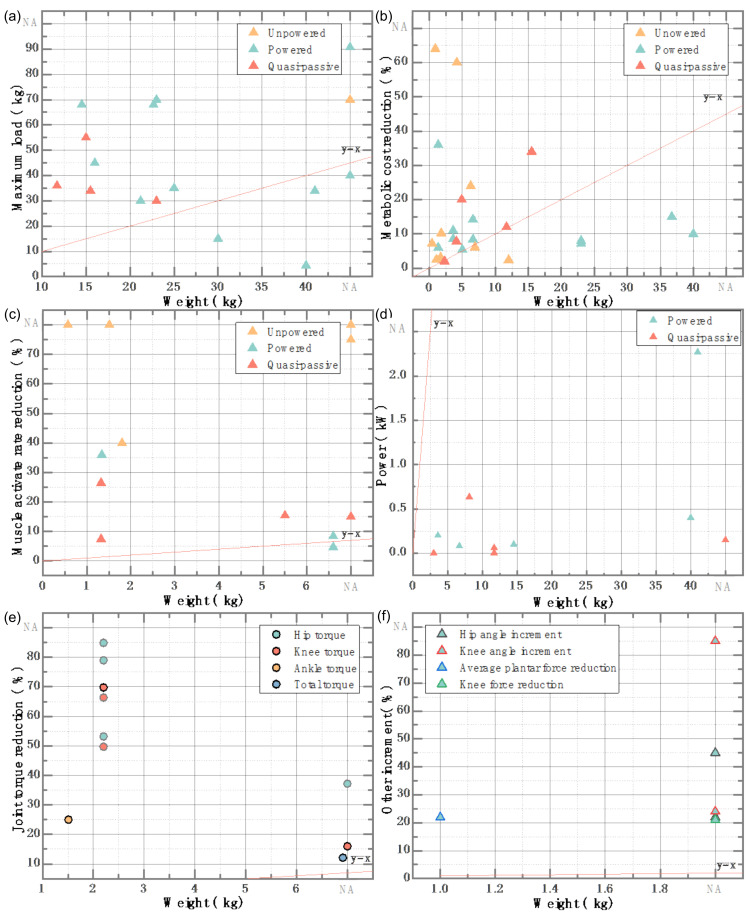
Relationship between weight and performance evaluation results. (**a**) maximum load with wearable exoskeleton. (**b**) the metabolic reduction with wearable exoskeleton, (**c**) muscle activate rate reduction with wearable exoskeleton, (**d**) device power for powered and quasi-passive exoskeleton, (**e**) joint toque reduction with the device, (**f**) other increment with wearable exoskeleton.

An obvious criterion for evaluation is the association between weight and the effectiveness of the assisted movement. The desired lower limb assisted exoskeleton has a high level of assisted performance, light mass, body comfort, and free motion. In this paper, different energy sources and elastic elements are used to assist in improving the ability to perform different human life activities, as shown in Figure 9. Additionally, the relationship between the weight of lower limb assisted exoskeleton and the evaluation of assist effect is shown in Figure 10.

The comparative analysis shows that wearable powered lower limb exoskeletons based on three types of actuators (motor drive, hydraulic drive, and pneumatic drive) are used more in load bearing and output (Figure 10a). However, the heavier equipment greatly interferes with human movement. Power-assisted exoskeleton based on elastic or flexible elements are more lightweight but have limited load capacity. The power-assisted effect is mainly reflected in the lower metabolism, muscle activation, and joint torque; the increased joint angle; and the decreased plantar pressure and joint force. In Figure 10, the linear equation $y=kx\left(k=1\right)$ is taken as the reference standard, where k represents the slope of the straight line. Make a straight line with the origin $\left(0,0\right)$ of the coordinate system through any point in the figure (exoskeleton evaluation index value). The greater the slope, the better the power-assisted effect of the exoskeleton device. Assisting the exoskeleton is a challenging task because it needs better coordination between its working state and body movement state.

Compared with the multi-joint unpowered exoskeleton robot, the single joint exoskeleton robot has lighter weight, smaller volume, and better assistance effect. This makes people’s acceptance of single joint exoskeleton robot higher than that of multi-joint exoskeleton robots. In addition, in some scenes, it is not necessary to assist multiple joints of the lower limb; specific joints can be assisted according to need.

### 4.2. Energy Storage Structure Design Based on Human Biomechanics

The design of the energy storage structure is the basis for power-assisted exoskeleton and directly determines the use effect of exoskeleton. The design should be based on the bionic configuration of the reproduction of the human motion spectrum, which can be adjusted according to the size of the user, and the design structure should be compact. Energy storage is the core element of the research and development on unpowered exoskeleton-assisted robots, and it is an important factor in their development. Table 8 describes the energy storage units of different wearable lower limb assistive exoskeletons.

Powered exoskeletons mostly use three energy forms: motor, hydraulic pressure, and air pressure. They refer to the biomechanical characteristics of human lower limbs in the process of movement, and they combine with a control strategy to achieve the effect of walking assistance and weight bearing. All elements are fixed on the exoskeleton and combined with the human body in the wearing of the devices.

Unpowered energy storage assisted exoskeletons can be used for daily walking assistance in hemiplegic patients and normal people. They serve as precise walking aids for paraplegic patients or normal people with different height, weights, and injuries. As a standard component of exoskeletons, the stiffness of energy storage spring is fixed. The selection of the stiffness parameters of the exoskeleton energy storage spring is an essential link in the research process. Exoskeletons with high stiffness will increase the discomfort of wearing, and the stiffness is insufficient for achieving the appropriate walking aid effect. The assist components used by the unpowered exoskeleton assist robots are usually a tension spring, torsion spring, pneumatic spring, constant force spring, scroll spring, single (double) spring, and spring lever and an elastic cable.

The quasi-passive exoskeleton is based on the passive (unpowered) exoskeletons with adjustable spring stiffness. These exoskeletons provide external support for the joints and help the patient stay standing. Through the compensatory effect of the patient’s residual muscles, the patient can realize walking. There is also the loss of energy by absorbing the muscle to do positive work, which can be used as the energy compensation when the muscle does negative work, so as to achieve the actual assistance effect.

The lower limb assisted exoskeletons using elastic/flexible energy storage elements usually need to be fixed on the waist and crotch, thigh, lower leg, and foot. Most of the elastic/flexible energy storage elements are located at the joints of the lower limbs and parallel to the muscles of the lower limbs. The bionic mechanical structure is used to simulate the movement of the lower limbs and to avoid disturbing the normal movement of the individual body. Finally, the wearable fixation makes it easy to attach and detach, making it more appropriate for long-term human wear.

### 4.3. Materials That Meet the Requirement of Wearable Exoskeleton Assistance

Assisted exoskeletons require high comprehensive properties of materials. Since heavy weight will lead to uncoordinated movement and high energy consumption [141], it is necessary to ensure that the material has high strength and low density and meets the use requirement of being lightweight. The exoskeleton material should be reasonably selected according to the design requirements and material characteristics of different parts. They should be adequate for wearers’ activities in complex environments and should reduce the impact of wearing quality on auxiliary effect.

The main materials of exoskeletons are titanium alloy, carbon fiber, acrylic plate, plastic, and aluminum alloy. So far, shape memory alloy is usually used as the assist component of unpowered exoskeleton assistive robots. With developments in material science, shape memory polymers, elastic matrix composites, multifunctional nanocomposites, liquid silica gel, and so on can now be used [142]. These materials form series elastic elements and variable stiffness elements singly or in combination [143], so as to further enrich the power-assisted elements of unpowered power assisted robot.

A multitude of textiles provide a sufficient amount of space for the wearable fixation of exoskeletons. There are various fabrics, including wool, cotton, and nylon, that can be used in wearable products. For example, large textiles, bandages with buckles, and nylon fasteners are used to keep the exoskeleton in immediate contact with the body.

### 4.4. Limitations and Challenges

Lower limb exoskeleton robots involve anatomy, bionics, ergonomics, mechanical transmission, material structure, and other disciplines. Although the design objectives and application scenarios of all kinds of lower limb exoskeleton robots will be quite different, their main design thrusts and precautions are similar [95]. The limitations and challenges faced in the development process are as follows: (1) material and structure innovation; (2) design of a muscle-like energy storage device; (3) design of an energy-switching device; (4) auxiliary effect evaluation; (5) man-machine cooperation; (6) control accuracy; and (7) comfort.

This paper systematically introduces and summarizes in detail the applicable objects, application scenarios, functional principles, mechanical structures, and assistance effects of exoskeleton assistance robots. It is helpful for readers to have a comprehensive and profound understanding of unpowered exoskeleton assisted robots. At present, there has been little research on unpowered exoskeleton robots, and as such, the technology is not yet mature. Therefore, in terms of unpowered drive, there are still some deficiencies, such as poor control accuracy, limited bearing capacity, poor man-machine coordination, and single types of actuators [144]. This will be the research focus and improvement direction in the future. We should develop products with powerful functions, high efficiency, strong adaptability, light weight and low cost. For the realization of applicable exoskeletons, it is imperative to realize the goal of integrating robots into our daily life [145].

## 5. Future Prospects

With the growing developments and advances in wearable lower limb assistive exoskeletons, their weights range from tens of kilograms to less than one kilogram, and their assistive performance has improved. One of the most common and complex activities of walking is constant energy consumption and transformation. Assisted exoskeletons aim to reduce the metabolic energy of human movement and enhance the load-bearing capacity of the human body. They are used for sports assistance, weight-bearing surgery, and assisted rehabilitation, maximizing the effect of the assistance and minimizing interference with the body’s normal movements. For the secondary utilization of human excess energy, we can try to further collect and store the energy lost in the process of human movement. It is used for emergency power supply of wearable electronic devices, life feature monitoring and assisted rehabilitation to minimize the waste of energy. There will be more and more exoskeleton robots with a positive cost of harvesting (COH).

The energy in the mechanical energy storage structure can assist human movement. However, the weight of the equipment itself causes the loss of metabolic energy, which is also very objective. Simple, compact, and lightweight materials have become important factors in reducing the weight and energy loss of exoskeletons. The research and development of unpowered exoskeleton has solved the energy and power problems that restrict the development of traditional powered exoskeleton to a certain extent. R&D has reduced the control difficulty and manufacturing costs in the process of robot research and development. It has also promoted the transformation of traditional weight-bearing applications and solved the problem of human assistance in many industries. With the rapid development of new structures, materials, processing and molding technologies, and detection techniques, wearable human lower limb assisted exoskeletons will develop rapidly. They have broad development prospects, strong market demand, and far-reaching application value.

The research and development of unpowered exoskeletons have solved the energy and power problems that previously restricted the development of traditional power-assisted exoskeletons to a certain extent.

The research and development of unpowered exoskeleton has solved the energy and power problems that restrict the development of traditional powered exoskeleton to a certain extent.

## Figures and Tables

**Table 1:** Motion range of lower limb joints.

Lower Limb Joint	Degree of Freedom (DOF)	Range of Motion	Limits of Motion
Hip joint	Internal/external rotation	1.6°/13.2°	50°/40°
	Flexion/extension	32.2°/22.5°	120°/30°
	Abduction/adduction	7.9°/6.4°	20°/45°
Knee joint	Flexion/extension	73.5°/0°	150°/0°
Ankle joint	Internal/external rotation	0°/0°	15°/15°
	Dorsiflexion/toe flexion	14.1°/20.6°	20°/40°
	Varus/valgus	16.5°/25.7°	20°/35°

**Table 2:** Powered lower limb multi-joint assisted exoskeletons.

Study	Year	Joint	App.	Weight & Load	Driver	Element	Subject	Speed
USA [46]	2004	H′ K′ A′	EWB	W (41); L (34)	Hydraulic actuator drive	P (2.27)	S′	1.3 m/s walking
USA [47]	——	H′ K′ A′	EWB	W (14.52); L (68.04)	Power supply and on-board computer	P (0.1)	——	2.5 MPH walking 42 miles
[47]	——	H′ K′ A′	EWB MA	W (22.68); L (68.04)	Power supply and on-board computer	——	——	Up 1000 feet
USA [48]	2006–2007	H′ K′ A′	——	W (3.6); L (68.04–90.72); L (36.74);	Hydraulic actuator drive	——	——	2 MPH walking
China [49,50]	2007	H′ K′ A′	EWB	W (21.2); L (30)	Air spring and motor drive	——	——	3.6 km/h walking 2 h
China [51,52,53]	2008	H′ K′ A′	MA	W (25); L (35)	Hydraulic drive	——	——	12 h walking
Japan [59]	2009	H′ K′ A′	EWB MA	W (23); L (70)	Motor drive	——	——	——
China [60]	2012	H′ K′ A′	EWB	W (30); L (15)	Hydraulic drive	——	1 M′	Stand upright
China [61,62]	2013	H′ K′	EWB	W (40); L (4)	Motor drive	P (0.4)	1 M′	——
USA [65]	2016	H′ K′ A′	MA	W (6.6); L (30% load for body weight)	Motor drive	——	7	1.5 m/s
China [63,64]	2016	H′ K′ A′	EWB	W (16); L (45)	Hydraulic drive	——	——	4 km/h walking 1 h

App.: Application.  H′: Hip; K′: Knee;  A′: Ankle;  F′: Foot; W: Weight (kg); L: Load (kg);  S′: Solider;  M′: Male;  F′: Female; HS: Heath subjects; SP: stroke patient; PSCI: patient with spinal cord injury; SM: simulation model; MA: Movement assistance; MR: Medical rehabilitation; ME: Motivation enhancement; EWB: Enhanced weight bearing; O: Orthosis; SC: Stiffness coefficient(N/mm); TS: Torsional stiffness (Nm/rad); P: Power (kW); ${\mathrm{MT}}_{m}$: Maximum torque of motor (Nm); ${\mathrm{MT}}_{d}$: Maximum torque of device (Nm);  P′: Normalized peak power (W/kg); P: Power (kW).

**Table 3:** Powered lower limb single-joint assisted exoskeletons.

Study	Year	Joint	App.	Weight & Load	Driver	Element	Subject	Speed
USA [66]	2016	H′	MA	W (23); L (23)	Hip suit and driving platform	——	8	1.5 m/s walking
USA [67]	2017	H′	MA	W (5)	Wearable system (drive and control)	——	8 M′	2.5 m/s running
USA [68]	2007	A′	MA	W (0.95)	Motor, lead screw, and spring	P′ (3.125) P (0.131)	2 HS (1 M′, 1 F′)	——
Belgium [35]	2013	A′	MA	W (1.34)	Pneumatic drive	——	——	——
[71]	2014	A′	MA	W (6.7)	Pneumatic muscle	P (0.0802)	——	——
USA [72]	2014	A′	MA	W (23); L (23)	Motor, gear, wire, and linkages	P′(2.3)	7	1.5 m/s level walking
USA [73]	2015	A′	MA	W (<0.88)	Motors, controllers, and Bowden cables	${\mathrm{MT}}_{d}$(121)	1 HS	——
USA [74]	2016	A′	MA	W (3.6)	Winch, motor, and an aluminum outer	P (0.2)	6 M′	1.4 m/s level walking

App.: Application.  H′: Hip; K′: Knee;  A′: Ankle;  F′: Foot; W: Weight (kg); L: Load (kg);  S′: Solider;  M′: Male;  F′: Female; HS: Heath subjects; SP: stroke patient; PSCI: patient with spinal cord injury; SM: simulation model; MA: Movement assistance; MR: Medical rehabilitation; ME: Motivation enhancement; EWB: Enhanced weight bearing; O: Orthosis; SC: Stiffness coefficient(N/mm); TS: Torsional stiffness (Nm/rad); P: Power (kW); ${\mathrm{MT}}_{m}$: Maximum torque of motor (Nm); ${\mathrm{MT}}_{d}$: Maximum torque of device (Nm);  P′: Normalized peak power (W/kg); P: Power (kW).

**Table 4:** Unpowered lower limb multi-joint assisted exoskeletons.

Study	Year	Joint	App.	Weight & Load	Element	Attribute	Subject	Speed
USA [78,79,80]	2006	H′ K′	MR MA	——	Two springs	——	6(5-HS, 1-SP)	0.447 m/s walking
USA [81]	2009	H′ K′ A′	MA	W (6.75) W (6.26)	Multi- & single-leaf fiberglass springs	——	9	Jump test
Holland [82]	2011	H′ K′ A′	MA	W (12)	Artificial tendon	——	9	1.11 m/s walking
Holland [83]	2014	H′ K′ A′	MA	W (6.91)	Artificial tendon	——	6(5 M′, 1 F′)	1.11 m/s walking
China [84]	2015	H′ K′ A′	MA EWB	W (2.357)	Torque spring and pressure spring	——	1	——
China [85]	2016	H′ K′ A′	MA	W (1)	Flexible double snap and trigger spring	——	5	COMSOL simulation
Canada [34]	2019	H′ K′ A′	MA	W (5.68)	Elastic wire	SC (0.58) SC (0.68) SC (0.97) SC (1.317) SC (1.734) SC (3.327)	1	Standing and walking test
China [86,87,88]	2019	H′ K′ A′	EWB MA	——	Spring	——	1	Walking on flat ground, climbing slopes and stairs
Canada [90]	2021	H′ K′ A′	MA	W (1.059)	Return spring	——	10-HS	Walking experiment
China [89]	2020	H′ K′	MR	W (2.2)	Pre-tensioned tension spring	——	5-HS	——

App.: Application.  H′: Hip; K′: Knee;  A′: Ankle;  F′: Foot; W: Weight (kg); L: Load (kg);  S′: Solider;  M′: Male;  F′: Female; HS: Heath subjects; SP: stroke patient; PSCI: patient with spinal cord injury; SM: simulation model; MA: Movement assistance; MR: Medical rehabilitation; ME: Motivation enhancement; EWB: Enhanced weight bearing; O: Orthosis; SC: Stiffness coefficient(N/mm); TS: Torsional stiffness (Nm/rad); P: Power (kW); ${\mathrm{MT}}_{m}$: Maximum torque of motor (Nm); ${\mathrm{MT}}_{d}$: Maximum torque of device (Nm);  P′: Normalized peak power (W/kg); P: Power (kW).

**Table 5:** Unpowered lower limb single-joint assisted exoskeletons.

Study	Year	Joint	App.	Weight & Load	Element	Attribute	Subject	Speed
Holland [91]	2016	H′	MA	W (4.15)	Leaf spring	——	1-HS	1 m/s; 1.25 m/s
China [92,93]	2016	H′	MR	——	Tension spring	——	1-PSCI M′	Stand and walking
Iran [94]	2018	H′	MA	W (1.8)	Bent leaf spring	——	10-HS	2.5 m/s running
Canada [95]	2012	K′	MR MA	W (0.9)	Liquid spring	——	——	——
USA [96]	2013	K′	MA	W (0.71)	Mechanical clutch and leaf spring	${\mathrm{MT}}_{m}$(190)	5	3.5 m/s running
China [97]	2017	K′	EWB	L (70)	Compression spring	——	5	1.36 m/s, 2.55 m/s
Korea [98]	2018	K′	EWB	L (10) L (20)	Four-link structure	——	1	2 km/h walking
China [99]	2018	K′	ME MR	W (1.07)	Torsion spring	P (0.2) P (0.225)	8-HS M′	30 km/h cycling
Sri Lank [100]	2018	K′	EWB	W (1.8)	Return spring	——	10-HS M′	1.3 m/s walking
China [101]	2019	K′	MA	W (1.7)	Scroll spring and generator	——	9-HS	3.3 km/h~5.4 km/h running
USA [103]	2011	A′	MR MA	W (0.57)	Linear tension spring	——	1	1.25 walking
USA [104]	2015	A′	MA	W (0.408-0.503)	Mechanical clutch and spring set	TS (180)	9	1.25 m/s walking
Canada [108]	2018	A′	MR ME	W (1.35)	Pneumatic artificial muscle	——	——	——
Canada [105,109]	2019	A′	MA	W (1.51)	Pneumatic artificial muscle	——	5	1.18 m/s; 1.17 m/s; 1.17 m/s; 1.16 m/s
China [107]	2019	A′	MA	——	Pre-tension spring	SC (25)	1	4 km/h

App.: Application.  H′: Hip; K′: Knee;  A′: Ankle;  F′: Foot; W: Weight (kg); L: Load (kg);  S′: Solider;  M′: Male;  F′: Female; HS: Heath subjects; SP: stroke patient; PSCI: patient with spinal cord injury; SM: simulation model; MA: Movement assistance; MR: Medical rehabilitation; ME: Motivation enhancement; EWB: Enhanced weight bearing; O: Orthosis; SC: Stiffness coefficient(N/mm); TS: Torsional stiffness (Nm/rad); P: Power (kW); ${\mathrm{MT}}_{m}$: Maximum torque of motor (Nm); ${\mathrm{MT}}_{d}$: Maximum torque of device (Nm);  P′: Normalized peak power (W/kg); P: Power (kW).

**Table 6:** Quasi-passive lower limb multi-joint assisted exoskeletons.

Study	Year	Joint	App.	Weight & Load	Element	Attribute	Subject	Speed
USA [110]	2005	H′ K′ A′	EWB	W (15.5); L (34)	Variable damper	——	1	0.82 m/s level walking
USA [111]	2006	H′ A′	MA	——	Springs, clutches, and variable damping	——	——	1.3 m/s level walking
USA [112]	2006	H′ K′ A′	EWB	W (15); L (20); L (40); L (55)	——	——	10 S	4.83 km/h level walking 8 min
USA [3]	2007	H′ K′ A′	MA EWB	W (11.7); L (36)	Variable damping and two springs	P (0.002)	1 M′	0.92 m/s walking
France [113]	2010	H′ K′ A′	MA EWB	——	Force balancer and driver	——	——	Ascend the stairs or slopes
USA [114]	2013	H′ K′ F′	O	W (3)	Actuator and spring module	P (0.0003) TS (109)	1	0.6 m/s walking
USA [115]	2014	H′ K′ F′	O	W (3)	Control module and support spring	TS (60)	3	——
Korea [116]	2014	H′ K′ A′	EWB	W (21); L (35)	Actuator and passive mechanism	${\mathrm{MT}}_{m}$(1.8422)TS (92.25)	1	6 km/h walking
China [117]	2015	H′ K′ A′	MA	——	Actuator and spring	——	——	——
Korea [118]	2015	H′ K′ A′	EWB MA	W (23); L (30)	Spring and driver	SC (69.6)	5 M′	4 km/h level walking; ascend the stairs
Japan [119]	2016	K′	MR ME	W (9)	Passive components and two drivers	${\mathrm{MT}}_{m}$ (100) TS (1000) ${\mathrm{MT}}_{d}$ (15)	——	Level walking, squat, stand to sit
Netherlands [120]	2018	H′ K′ A′	EWB MA	W (21.8)	Hydraulic piston damping	——	9	4.5~5.3 km/h walking
Italian [121]	2018	H′ K′	MA MR	W (4.1)	Elastic belt and clutch	——	1-SP M′	——
Italian [122]	2019	H′ K′	MA MR	W (4.9)	Elastic belt and clutch	——	1-SP M′	10 straight walks
Italian [123]	2020	H′ K′ A′	MA	W (4)	Pressure sensor, solenoid valve, and vacuum generator	——	——	(2) Walk a straight path of 10 m with 10 min.

App.: Application.  H′: Hip; K′: Knee;  A′: Ankle;  F′: Foot; W: Weight (kg); L: Load (kg);  S′: Solider;  M′: Male;  F′: Female; HS: Heath subjects; SP: stroke patient; PSCI: patient with spinal cord injury; SM: simulation model; MA: Movement assistance; MR: Medical rehabilitation; ME: Motivation enhancement; EWB: Enhanced weight bearing; O: Orthosis; SC: Stiffness coefficient(N/mm); TS: Torsional stiffness (Nm/rad); P: Power (kW); ${\mathrm{MT}}_{m}$: Maximum torque of motor (Nm); ${\mathrm{MT}}_{d}$: Maximum torque of device (Nm);  P′: Normalized peak power (W/kg); P: Power (kW).

**Table 7:** Quasi-passive single-joint assisted lower limb exoskeletons.

Study	Year	Joint	App.	Weight & Load	Element	Attribute	Subject	Speed
Slovenia [126]	2020	H′	MA	W (1.75)	Clutch, servo motor, encoder, and spring	——	7-HS	Walk, sit, squat and lift load randomly
USA [127]	2004	K′	MA EWB	W (< 8.13)	Series elastic actuator	P (0.634)	——	——
Canada [128]	2008	K′	MA	W (11.7); L (36)	Spring, clutch, and variable damping	P (0.06)	1	Optional speed walking
USA [129]	2012	K′	MA	W (2.31)	Leaf spring and clutch	TS (3.6)	6	3.2 m/s running
Italian [130]	2014	K′	MA	——	——	TS (200) ${\mathrm{MT}}_{d}$ (45)	——	Standing and sitting
USA [131]	2014	——	——	——	——	0%, 33%, 66%, and 100% of the knee stiffness	7	Walking
USA [132]	2014	K′	MA	W (2.63); W (2.45);	Motor, worm gear set, and spring	——	——	4.83 km/h walking
Italian [133]	2016	K′	MR ME	——	——	——	——	——
USA [134]	2017	K′	MR	W (0.76)	Two outboard motors	${\mathrm{MT}}_{d}$ (25)	1 M′	1.25 m/s walking
USA [135]	2017	K′	MA		Air spring and solenoid valve	——	1 F′	Walk at a speed of 1m/s with a slope of −5°
USA [5]	2018	K′	MA	W (3.2)	Gear and 1-DOF rolling joint	——	3-HS	——
Italian [136]	2021	K′	ME	W (5.5)	Soft clutch and elastic belt	${\mathrm{MT}}_{d}$ (12)	1-HS	3 km/h walking
China [137]	2015	A′	MR MA	——	Motor, clutch, and ball screw	SC (17.97) P (0.15) ${\mathrm{MT}}_{d}$ (15)	1-SM M′	1.0 m/s walking
USA [138]	2020	A′	MA	W (1.323)	Motor and slider	SC (5.8) TS (169–362)	1-HS	1.0 m/s & 1.5 m/s walking
Slovenian [139]	2020	A′	MA	W (1.09)	Ratchet and pawl clutch	——	7-HS M′	Walking

App.: Application.  H′: Hip; K′: Knee;  A′: Ankle;  F′: Foot; W: Weight (kg); L: Load (kg);  S′: Solider;  M′: Male;  F′: Female; HS: Heath subjects; SP: stroke patient; PSCI: patient with spinal cord injury; SM: simulation model; MA: Movement assistance; MR: Medical rehabilitation; ME: Motivation enhancement; EWB: Enhanced weight bearing; O: Orthosis; SC: Stiffness coefficient(N/mm); TS: Torsional stiffness (Nm/rad); P: Power (kW); ${\mathrm{MT}}_{m}$: Maximum torque of motor (Nm); ${\mathrm{MT}}_{d}$: Maximum torque of device (Nm);  P′: Normalized peak power (W/kg); P: Power (kW).

**Table 8:** The advantages and disadvantages of lower limb assisted exoskeletons.

Type	Energy Storage Element	Advantage	Disadvantage
Powered lower limb assisted exoskeleton	Motor drive	(1) Rapid response and high sensitivity; (2) Easy automatic control; (3) No pollution and low noise; (4) High control precision; (5) High reliability and convenient maintenance	(1) Difficult to control directly and the reducer needs to be connected; (2) Low energy mass ratio; (3) Large inertia and slow steering; (4) Poor cushioning
	Hydraulic drive	(1) High energy mass ratio; (2) Simple structure and small inertia; (3) Easy to realize overload protection; (4) Driven directly; (5) Good buffer; (6) Easy to realize stepless speed regulation	(1) The working fluid is easy to leak and inconvenient to maintain; (2) The oil is easy to be compressed, and the control accuracy is general; (3) Polluted and noisy.
	Pneumatic drive	(1) Driven directly; (2) Low cost; (3) Easy to realize stepless speed regulation; (4) No pollution; (5) Simple system with reliable components; (6) Good buffer	(1) Low energy mass ratio; (2) High noise and easy air leakage; (3) Low control accuracy; (4) Poor structural stiffness; (5) Slow energy transfer and large delay
Unpowered lower limb assisted exoskeleton	Spring energy storage	(1) Lightweight and small volume; (2) Certain gravity compensation; (3) Greatly reduced metabolic energy consumption; (4) Certain support assistance; (5) Hardly affects normal gait	(1) The application scene is limited and interferes with the gait of other scenes; (2) Instability; (3) The spring with large stiffness coefficient makes the wearer feel uncomfortable; (4) The storage and release nodes of energy in gait cycle are fixed; (5) Higher torque is not suitable for patients who need rehabilitation
	Pneumatic muscle	(1) Adjustable binding force; (2) Higher matching with human muscles; (3) does not affect the free movement of the joint in the swing phase	(1) The timing mechanism needs lubricant added periodically to ensure its correct operation; (2) Device displacement
	Four-link structure	(1) High material strength; (2) Springs and damping elements are not used	The auxiliary effect is small at zero load or small load
	Adjustable artificial tendon	No energy switching device is required	(1) High quality; (2) Affect normal gait; (3) The wearer will have some discomfort
